# Choose Wisely: Great Variation among Genotypes of Promising Paludiculture Crop *Phragmites australis*

**DOI:** 10.3390/plants12051045

**Published:** 2023-02-24

**Authors:** Kerstin Haldan, Kristina Kuprina, Meike Ingeborg Haase, Fabian Kieckhäfer, Lisa Schade, Joraine Schmoldt, Lina Stella Schock, Marthe Stein, Alexander Wille, Martin Schnittler, Manuela Bog, Jürgen Kreyling

**Affiliations:** Institute of Botany and Landscape Ecology, University of Greifswald, Partner in the Greifswald Mire Centre, 17489 Greifswald, Germany

**Keywords:** *Phragmites australis* genotypes, common reed, paludiculture, functional traits, gene expression, plant strategies, mesocosm gradient experiment, RT-qPCR

## Abstract

Measures mitigating the climate crisis, such as paludiculture, which is the agriculture on rewetted peatlands, are urgently needed. The cosmopolitan species *Phragmites australis* has the potential to be used in paludiculture worldwide but is known for its high intraspecific variation. This raises the questions of whether (i) *P. australis* genotypes differ even at a regional scale, making them differently well suited for paludiculture and (ii) *P. australis* performance can be predicted by linking the variation in genotypes to strategies in the plant economics spectrum. Five *P. australis* genotypes from Mecklenburg-Western Pomerania were cultivated in two 10-month mesocosm experiments along gradients of water level and nutrient addition. We compared growth, morphology (height, growing density), above- and belowground biomass, functional and ecophysiological traits (SLA, LDMC, SRL, RDMC, root porosity, photosynthetic rate) as well as gene expression. Our results demonstrate a high variability of *P. australis* genotypes even at a regional scale, revealing genotype-specific productivity, morphology, and gene expression and implying that the selection of suitable genotypes will be crucial for the success of a paludiculture. However, trait covariation did not indicate distinct plant economic strategies to predict genotype performance. Instead, large-scale genotype trials are needed to select suitable genotypes for paludiculture.

## 1. Introduction

Peatlands are highly effective carbon sinks—while covering only about 3% of the Earth’s land surface, they are the largest terrestrial carbon store [1,2,3]. Degraded peatlands, however, are responsible for approximately 2 Gt of CO_2_ emissions per year due to peat oxidation upon drainage [4]. Furthermore, drainage of peatlands diminishes their water and nutrient retention function, leading to soil subsidence which requires ever-deeper drainage for continued agriculture [5]. Considering these consequences of peatland drainage, large-scale rewetting of peatlands is inevitable [6,7]. Paludiculture, the agriculture on wet or rewetted peatlands, provides an alternative to conventional drainage-based agriculture. It combines productive use with other ecosystem services of wet peatlands, such as sequestering and storing carbon in peat, water retention and purification, retention of nutrients and pollutants, microclimate regulation, and habitat for specifically adapted organisms [8,9]. A plant species that is especially well suited for paludiculture is the common reed, *Phragmites australis* (Cav.) Steud. It is a cosmopolitan species that occurs between 70° N and 43° S on every continent except Antarctica, in temperate, tropical, and even arid regions [10]. Introduced populations in Australia and North America are naturalized and can be invasive [10]. *P. australis* often forms dense, monodominant stands on the banks of lakes, rivers, and ditches, in peatlands and freshwater to brackish swamps [11,12]. The species’ growth and performance are negatively influenced by increasing salinity, but genotypes exist that can tolerate a salinity of up to 65‰ in soil pore water [13,14]. Hydrology is a strong driver of *P. australis* occurrence; it has a wide ecological amplitude (water table −6 to 2.3 m) but grows best at a water table of −30 to +70 cm [10] and can tolerate constantly wet conditions, under which it is peat-forming [15]. While *P. australis* can grow under very low nutrient availability, it prefers nutrient-rich habitats where it is highly productive [16,17,18].

Cultivation of *P. australis* in a paludiculture serves multiple goals: The aboveground biomass offers a wide range of utilization options; it can be used as roof thatching material [19], building and insulation material [20], bioenergy feedstock [21], or for paper production [22]. The belowground biomass is peat-forming and thereby acts as a long-term carbon store. Fen peat is mainly formed by roots and rhizomes growing into an existing peat matrix (‘displacement peat’), with their decomposition being strongly reduced under water saturation [15,23,24]. *P. australis* is the main peat-forming species in plant communities, such as reed stands (*Schoenoplecto-Phragmitetum*) and sedge communities (*Phragmiti-Magnocaricetea)* [25,26].

*P. australis* is a clonal plant with high intraspecific variability [27,28]. Genotypes differ in productivity [29,30] and morphological characteristics such as growth density, stem height and diameter, and leaf size and shape [28,29,30,31]. These differences may be genetically determined and genotype-dependent as they persist when genotypes are transplanted [27,28,29,30]. However, the species can show high plasticity in response to environmental disturbances [32]. *P. australis* occurs in different ploidy levels, and some studies report ploidy-dependent morphological characteristics and tolerances [28,33,34], while others show considerably higher variation between genotypes than ploidy levels or geographical origins [14,27]. Stress response in *P. australis* can be highly genotype-specific, indicated, for example, by different expression of genes involved in photosynthesis, oxidative stress response, and Na+/H+-transport [27,35,36,37].

*P. australis* genotypes may follow different strategies described by [30,38]. The ’assimilation type’ stems from a nutrient-rich habitat, is characterized by high biomass N-content, and relocates only little N to rhizome storage, producing a highly decomposable litter. Contrary, the ’translocation type’ stems from a nutrient-limited site and shows effective translocation of N to storage organs with little N remaining in its litter. The assimilation and translocation strategies can be viewed as one aspect of the ‘plant economics spectrum’, with ‘fast’ or ‘slow’ strategies across plant organs [39,40,41,42].

Linked to a ‘fast’ strategy are a high photosynthetic rate, high specific leaf area (SLA) and specific root length (SRL), low leaf (LDMC) and root dry matter content (RDMC), high root and low rhizome mass fraction and low N content in winter aboveground biomass [38,39,41,42,43]. These traits lead to a high resource acquisition capacity above- and belowground, consequently to a high potential growth rate [39,40] and high productivity [38]. The ‘slow’ strategy is described by opposite characteristics in the above-mentioned traits, resulting in a low potential growth rate and low productivity [38]. The ’fast’ strategy should be advantageous under favorable conditions but is not tolerant of low resource availability, resulting in a bad performance under stressful conditions [40]. However, the low resource requirements of the ‘slow’ strategy are advantageous under nutrient-poor conditions and result in better performance under resource limitation but less or no increase in performance towards favorable conditions [40]. Evidence of plants following different strategies has been found to occur within species [44], e.g., *P. australis* [45], and even within clones of a species [46].

If *P. australis* genotypes differ in their performance, the question arises of how to select suitable genotypes for paludiculture. Here, we investigate whether plant strategies can be used to predict the performance of genotypes. In our study, five *P. australis* genotypes from thatching reed stands in northeast Germany were cultivated in gradients of resource availability (nutrients, water) and their productivity (above- and belowground biomass), morphology (height, growing density), functional traits (SLA, SRL, LDMC, RDMC, shoot elongation rate, photosynthetic rate, root porosity, biomass allocation), and gene expression were assessed to investigate the following hypotheses:

(1) *P. australis* genotypes differ in biomass productivity, and morphological traits and are thus differently well suited for paludiculture;
(2) Conditions at the extreme ends of the resource availability gradients (macronutrient deficiency/surplus; drought/flooding) cause stress for *P. australis* and lead to a higher expression of oxidative stress response genes;
(3) Differences in productivity and growth among *P. australis* genotypes arise from the fact that they follow different strategies in the plant economics spectrum and are indicated by (a) ‘fast’ genotypes outperforming ‘slow’ genotypes under favorable conditions and suffering stronger under stressful conditions, and (b) functional trait differentiation between genotypes according to these plant strategies;
(4) Performance of *P. australis* genotypes for paludiculture can be predicted using functional traits and plant strategies.

## 2. Results

**Figure 1:**
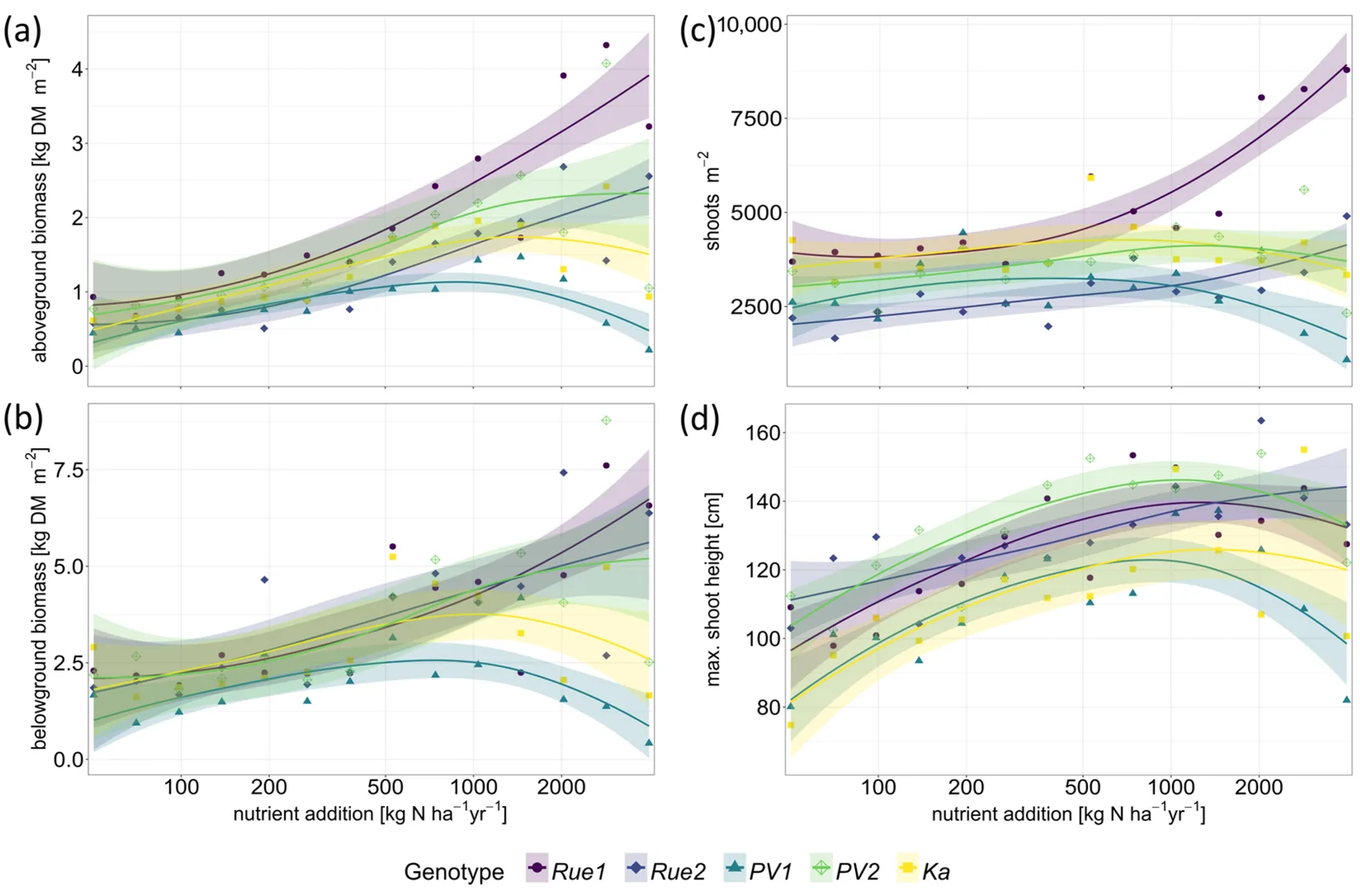
Biomass and morphology of five *P. australis* genotypes (*Rue1*, *Rue2*, *PV1*, *PV2*, *Ka*; Table 1) along the nutrient addition gradient. (**a**) Aboveground biomass dry weight [kg m^−2^] (span = 1.5) and (**b**) belowground biomass dry weight [kg m^−2^] (span = 1.5) at harvest in February 2020. (**c**) Number of shoots per m^2^ (span = 1.5) and (**d**) maximum shoot height [cm] (span = 1.8) at the end of the growing season on 18 September 2019. Symbols show original data points; lines are the smoothed local polynomial regression fittings (loess). Shaded areas around lines indicate 83% confidence intervals.

**Figure 2:**
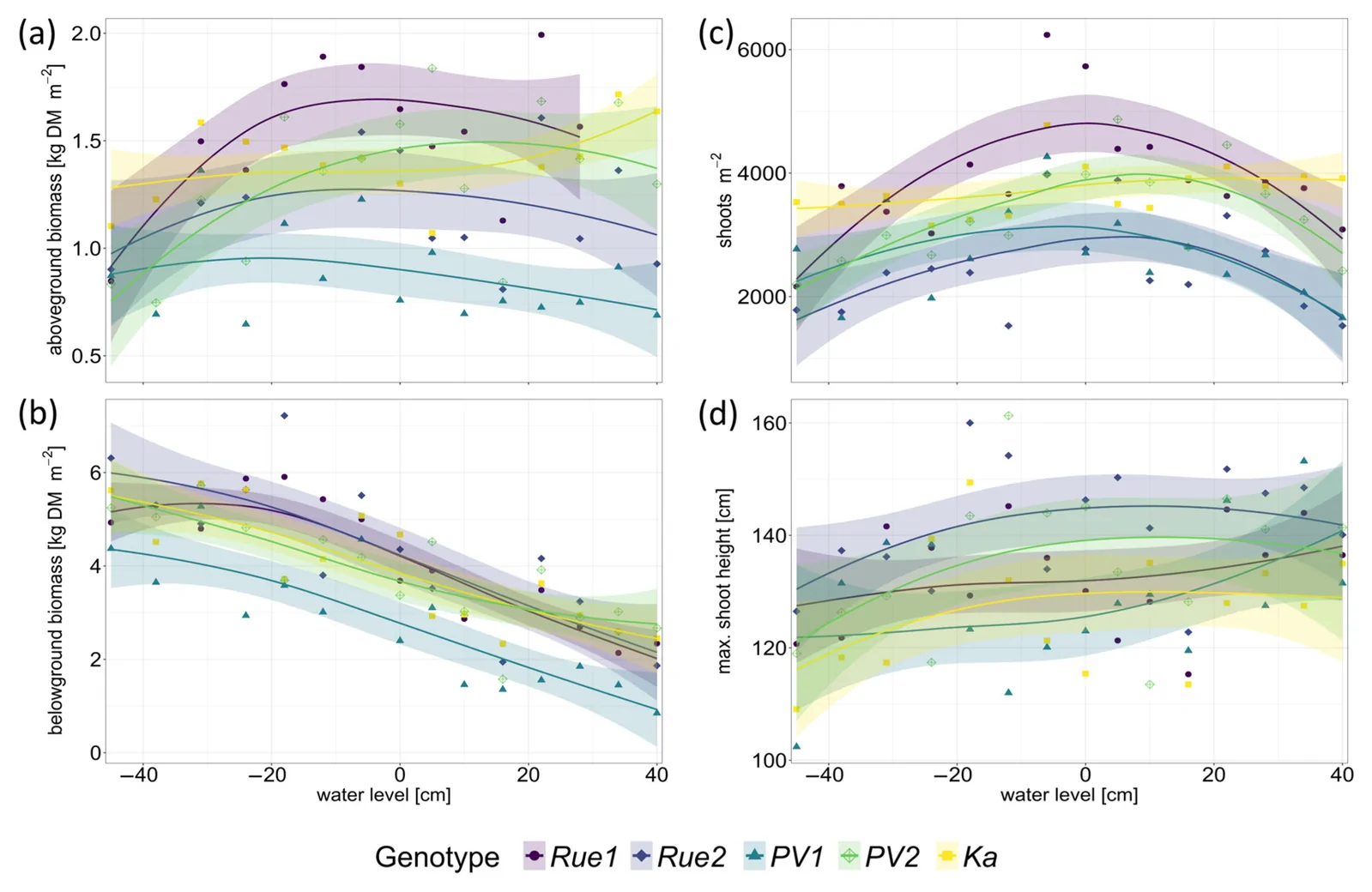
Biomass and morphology of five *P. australis* genotypes (*Rue1*, *Rue2*, *PV1*, *PV2*, *Ka*; Table 1) along the water level gradient. (**a**) Aboveground biomass dry weight [kg m^−2^] (span = 1.7) and (**b**) belowground biomass dry weight [kg m^−2^] (span = 1.4) at harvest in February 2020. (**c**) Number of shoots per m^2^ (span = 1.2) and (**d**) maximum shoot height [cm] (span = 2.5) at the end of the growing season on 18 September 2019. Negative numbers are water levels below ground, and positive numbers are water levels above ground. Symbols show original data points; lines are the smoothed local polynomial regression fittings (loess). Shaded areas around lines indicate 83% confidence intervals.

Five *P. australis* genotypes (*Rue1*, *Rue2*, *PV1*, *PV2* and *Ka*; Table 1) were studied. Genotypes differed in aboveground and root biomass production (Figure 1a,b, Figure 2a,b, and Figure S1) and showed less pronounced differences in rhizome biomass (Figure S1). All genotypes were similar in biomass production at low water levels and low nutrient additions, respectively, and differences were more pronounced at high water levels and high nutrient additions (Figure 1a,b and Figure 2a,b). Along the nutrient addition gradient, significant differences between genotypes in above- and belowground biomass occurred upwards of an addition of approximately 500 kg N ha^−1^ yr^−1^. Along the water level gradient, significant differences between genotypes in aboveground biomass occurred at water levels higher than 20 cm below ground; in belowground biomass, only genotype *PV1* differed significantly from the other genotypes along almost the entire gradient. On average, *Rue1* was the most productive genotype regarding total biomass, and *PV1* was the least productive. More productive genotypes (*Rue1*, *PV2*) had their optimum productivity at higher nutrient addition along the nutrient addition gradient compared to less productive genotypes (*PV1;* Figure 1a,b). Aboveground biomass increased with increasing nutrient addition for all genotypes and belowground biomass for genotypes *Rue1*, *Rue2,* and *PV2*. However, above- and belowground biomass responded differently to the water level gradient: with increasing water level, belowground biomass of all genotypes decreased while aboveground biomass of *PV2* increased and aboveground biomass of the other genotypes was not significantly affected by water level (Figure 2a,b). The theoretical aboveground yield of genotypes *Rue1* and *PV1* differed 1.6-fold (6.20 t ha^−1^ to 9.95 t ha^−1^; nutrient addition gradient) to twofold (8.37 t ha^−1^ to 16.22 t ha^−1^; water level gradient) under likely paludiculture conditions. Under the same conditions, belowground yield differed 1.4-fold between genotypes *PV1* (16.39 t ha^−1^) and *Ka* (23.23 t ha^−1^) in the nutrient addition gradient and 1.7-fold between genotypes *PV1* (20.77 t ha^−1^) and Rue 2 (34.37 t ha^−1^) in the water level gradient. Notably, the most productive genotypes regarding belowground yield under assumed paludiculture conditions were *Ka* in the nutrient addition gradient and *Rue2* in the water level gradient, and not the overall most productive genotype *Rue1*. Differences between genotypes in theoretical above- and belowground yield under assumed paludiculture conditions were not significant at *p* ≤ 0.05.

The genotypes differed in morphology (Figure 1c,d and Figure 2c,d). Averaged over the gradients, *Rue1* had many shoots and grew to a medium height (mean number of shoots in the water level/nutrient addition gradient: 127/162; mean height in the water level/nutrient addition gradient: 132/127 cm); *Rue2* produced few shoots but grew tall (78/90; 142/129 cm); *PV1* also produced few shoots but stayed small (86/90; 128/110 cm); *PV2* produced a medium amount of shoots and grew tall (105/115; 135/134 cm); *Ka* also produced a medium amount of shoots but stayed small (117/125; 127/125 cm). Increasing nutrient addition led to an increase in maximum plant height in all genotypes, while water level had no significant effect. The number of shoots increased with increasing nutrient addition in genotypes *Rue1* and *Rue2*. All genotypes except *Ka* showed a trend of optimum shoot number at water levels close to the soil surface; the decrease of shoot number from water levels at the soil surface to lower water levels was significant in genotypes *Rue1* and *PV2*, the decrease towards higher water levels was significant in genotype *PV2*.

The shoot elongation rate decreased towards the end of the growing season without consistent differences over time and along the gradients between the genotypes (Figure S2).

Belowground mass fraction tended to decrease with increasing nutrient addition and did significantly so for genotype *Ka*, while it decreased significantly towards high water levels in all genotypes (Figure S3a,d). Overall, *Rue1* had a low (water level/nutrient addition gradient: 0.736/0.652), and *Rue2* had a high belowground mass fraction (water level/nutrient addition gradient: 0.768/0.739) compared to the other genotypes. Averaged over each gradient, respectively, *PV1* showed a low root (water level/nutrient addition gradient: 0.231/0.186) and high rhizome mass fraction (0.508/0.508), while *Rue1* had a high root (0.377/0.321) and low rhizome mass fraction (0.358/0.331) compared to the other genotypes (Figure S3b,c,e,f).

The photosynthetic rate decreased with increasing water level for genotypes *Rue1*, *Rue2*, *Ka*, and *PV1* and increased at high nutrient addition in genotypes *Rue1*, *PV1,* and *PV2* (Figure S4).

The root porosity of all genotypes increased with increasing water levels (Figure S5b). Along the nutrient addition gradient, the genotypes differed (Figure S5a): while the root porosity of *Ka*, *PV1,* and *PV2* increased with increasing nutrient addition, the root porosity of *Rue1* and *Rue2* followed an optimum curve. Averaged over the nutrient addition gradient, *Ka* had the lowest (15.3%) and *Rue2* the highest (22.1%) root porosity; averaged over the water level gradient, however, *Rue2* had the lowest (13.9%) and *PV1* the highest (24.9%) root porosity.

Specific leaf area (SLA) decreased with increasing nutrient addition in all genotypes and increased with the increasing water level in *Rue1*, *Ka*, *PV1,* and *PV2*, but not *Rue2* (Figure S6a,c). Averaged over each gradient, respectively, SLA was lower in *Rue1* and *Rue2* (water level/nutrient addition gradient *Rue1* 22.7/22.5 m^2^ kg^−1^; *Rue2* 22.4/22.5 m^2^ kg^−1^) compared to *Ka*, *PV1,* and *PV2* (*Ka* 23.2/23.9 m^2^ kg^−1^; *PV1* 24.6/24.4 m^2^ kg^−1^; *PV2* 24.8/23.7 m^2^ kg^−1^). *PV1* and *PV2* exhibited a higher SLA compared to the other genotypes at water levels above the soil surface. *PV1* and *PV2* also exhibited a lower leaf dry matter content (LDMC) than *Rue1* and *Rue2* across both gradients (Figure S6b,d; mean LDMC in water level/nutrient addition gradient: *Rue1* 384/390 mg g^−1^; *Rue2* 392/398 mg g^−1^; *Ka* 380/371 mg g^−1^; *PV1* 351/363 mg g^−1^; *PV2* 347/363 mg g^−1^).

The root traits specific root length (SRL), root dry matter content (RDMC), and root diameter did not reveal a consistent pattern of differences between the genotypes. Notably, there was very little differentiation between genotypes in the water level gradient (Figure S7). SRL tended to increase with increasing water level, significantly in genotype *PV1*. SRL decreased with increasing nutrient addition in every genotype, with *PV1* showing an initial increase before the decrease.

**Figure 3:**
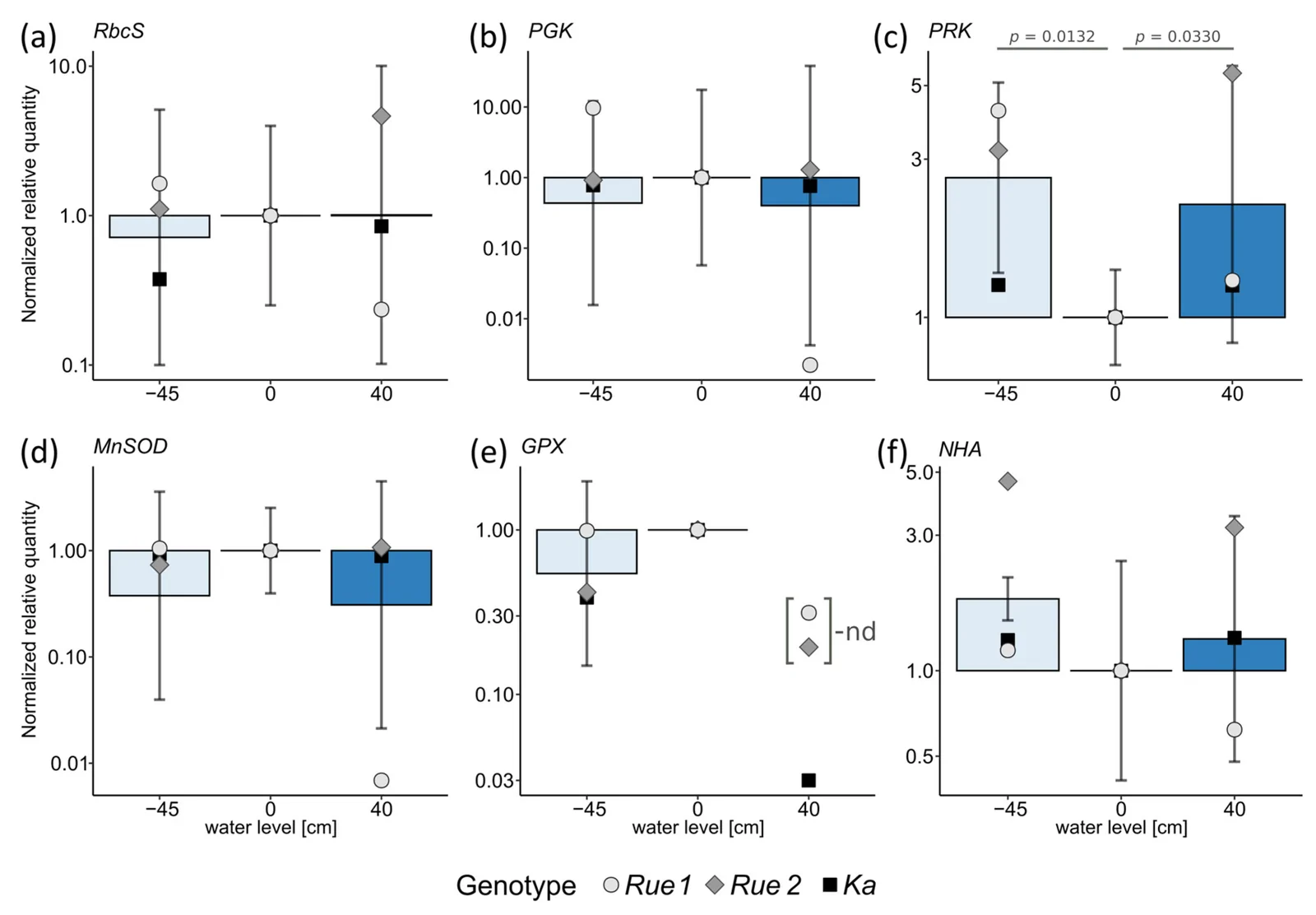
Normalized relative quantity of three photosynthetic genes ((**a**) *RbcS*, Ribulose bisphosphate carboxylase small chain, (**b**) *PGK*, Phosphoglycerate kinase, (**c**) *PRK*, Phosphoribulokinase), two oxidative stress response genes ((**d**) *MnSOD*, Manganese superoxide dismutase, (**e**) *GPX*, Glutathione peroxidase), and one transporter gene ((**f**) *NHA*, Na+/H+ antiporter) of three genotypes of *P. australis* (*Rue1*, *Rue2*, *Ka*; Table 1) at three water levels. Bar plots and error bars are built for the standardized data. Error bars show 95% confidence intervals, *p*-values (BH corrected) are given for pairs with statistically significant t-test results, nd—not determined (low expression).

**Figure 4:**
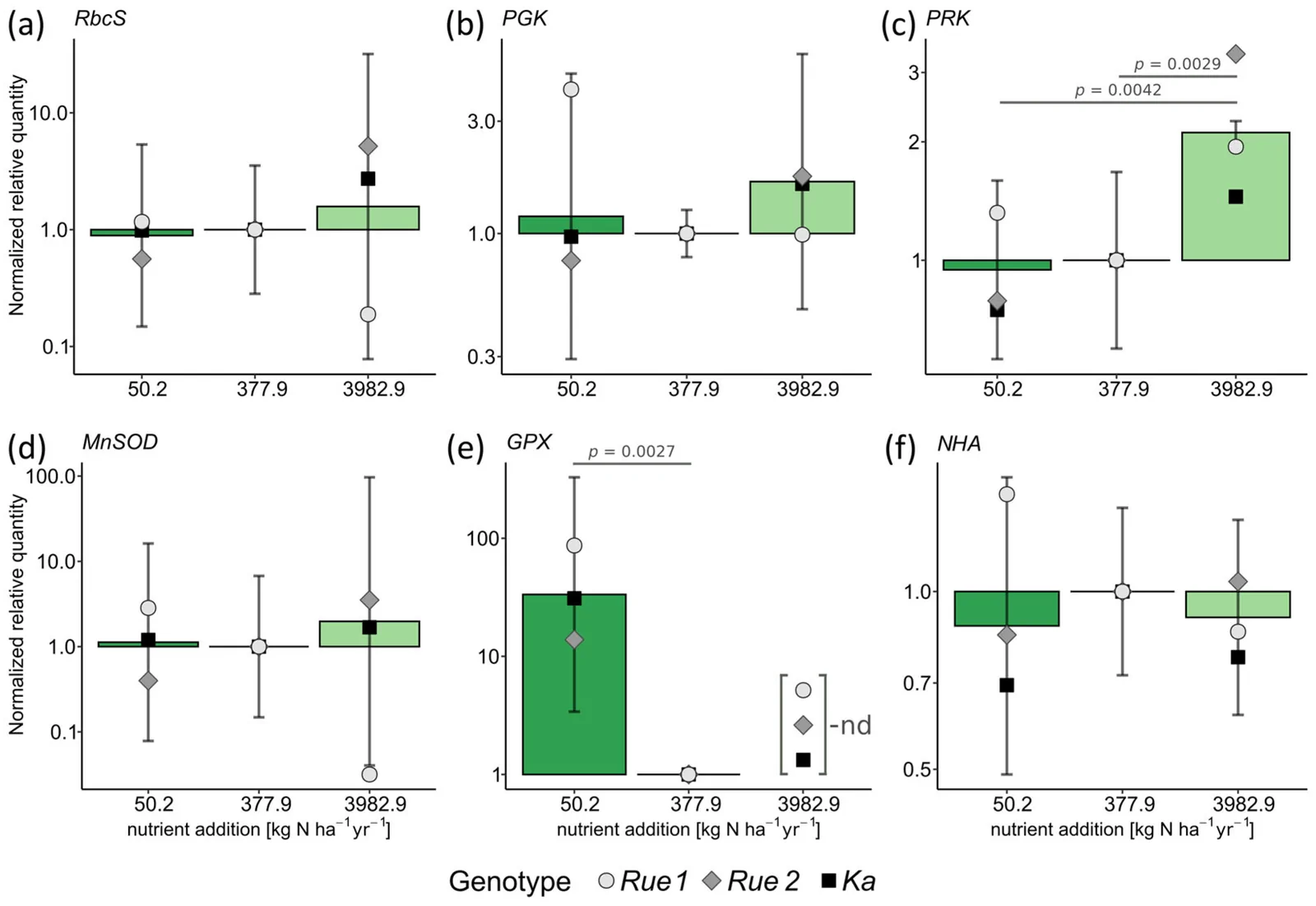
Normalized relative quantity of three photosynthetic genes ((**a**) *RbcS*, Ribulose bisphosphate carboxylase small chain, (**b**) *PGK*, Phosphoglycerate kinase, (**c**) *PRK*, Phosphoribulokinase), two oxidative stress response genes ((**d**) *MnSOD*, Manganese superoxide dismutase, (**e**) *GPX*, Glutathione peroxidase), and one transporter gene ((**f**) *NHA*, Na^+^/H^+^ antiporter) of three genotypes of *P. australis* (*Rue1*, *Rue2*, *Ka*; Table 1) at three levels of nutrient addition. Bar plots and error bars are built for the standardized data. Error bars show 95% confidence intervals, *p*-values (BH corrected) are given for pairs with statistically significant t-test results, nd—not determined (low expression).

Expression of *PRK* and *GPX* was similarly regulated for the studied genotypes (*Rue1*, *Rue2*, *Ka*) (Figure 3 and Figure 4). *PRK* was upregulated at low (NRQ = 2.88; *p* = 0.0132) and high (NRQ = 2.66; *p* = 0.0330) water levels and upregulated at the high nutrient level (NRQ = 2.25; *p* = 0.0042). *GPX* was downregulated at the high water and nutrient levels (below the detection limit) and highly upregulated (NRQ = 43.91; *p* = 0.0027) at the low nutrient level. The other genes (*RbcS*, *PGK*, *MnSOD*, *NHA*) showed different regulation patterns for studied genotypes (Figure 3 and Figure 4). The genes tended to decrease the expression with the increase of water level and nutrient addition in *Rue1*, while in genotype *Rue2, RbcS* was upregulated at high water and nutrient levels, and *NHA* was upregulated at drought and flooding. In genotype *Ka*, the expression of these genes did not change substantially at the extreme ends of the water and nutrient gradients.

## 3. Discussion

### 3.1. Genotypes Differ in Productivity and Morphology

The studied *P. australis* genotypes differed in their above- and belowground productivity and morphology, as assumed in hypothesis 1. This insight is highly relevant for paludiculture, as aboveground biomass provides income for the farmer, and belowground biomass forms peat [18]. Aboveground yield differed, although not significantly, up to twofold between the most and least productive genotypes in ranges of the gradients, which are realistic under field conditions (‘assumed paludiculture conditions’: 60 to 80 kg N ha^−1^ yr^−1^ nutrient addition [47] and 0 to +30 cm water level [48]). Genotypes differed even more, up to approximately eightfold, in aboveground biomass production at extremely high nutrient addition, which goes beyond what one would expect under unfertilized field conditions, but may be encountered in treatment wetlands [47,49,50,51]. Although calculated yields from experimental conditions should not be regarded as yields actually achievable under field conditions, these differences impressively demonstrate the high variability among *P. australis* genotypes. It can be expected that differences in the productivity of all genotypes in a region or in the whole range of the species are even higher [17]. The high variance in productivity among the genotypes in our study ties in with results of previous studies on *P. australis* genotypes from a similarly small-scale area: two *P. australis* genotypes from east Germany differed up to sevenfold in their aboveground biomass [38] while other *P. australis* genotypes from northeast Germany differed nearly threefold in their aboveground and 1.5-fold in their belowground productivity [29,30]. Similarly, our study showed that belowground productivity differed up to 1.7-fold between genotypes under assumed paludiculture conditions, and even more under extremely high nutrient addition. Since productivity is one of the main factors influencing peat formation [24,52], this indicates that the studied genotypes have different peat-forming potentials. This study only considered productivity, not the decomposition of *P. australis* belowground biomass. Belowground biomass of *P. australis* decomposes more slowly than that of other peat-forming species, e.g., *Carex* spp.; *P. australis* can therefore be considered to have a high peat-forming potential [15].

Differences in productivity and morphology of *P. australis* genotypes remain after transplanting into new environments [27,28,29,30]. In our study, the genotypes differed in height and shoot density, and while these traits were partly influenced by nutrient addition and water level, the genotypes kept their differences in relation to each other [29,53] with implications for subsequent use. For example, a certain culm length and diameter is preferred for thatching reed [54,55,56], which is an established and profitable utilization option [57]. Further biomass properties, which were not part of this study, must be considered for other utilizations. For energetic use or paper production, the chemical composition of biomass is important and has been shown to differ between *P. australis* genotypes [55,58]. Our findings demonstrate the importance of selecting appropriate *P. australis* genotypes regarding the aims of paludiculture.

### 3.2. Functional Traits and Performance along Gradients of Resource Availability Do Not Reveal Different Plant Strategies

*Rue1* and *PV1* differed most in total and aboveground productivity. Consequently, the high productive genotype *Rue1* would be at the ‘fast’ end and the less productive genotype *PV1* at the ‘slow’ end of the plant economics spectrum.

To investigate a differential performance of plants along gradients of resource availability, these need to provide a range of favorable to stressful conditions [40]. At the extreme ends of the water availability gradient, −45 cm and 40 cm, expression of genes related to oxidative stress *MnSOD* and *GPX* was either not significantly different from the expression at 0 cm water level or was even downregulated, revealing the absence of oxidative stress and indicating that the highest and lowest water levels in our study were not stressful for *P. australis* [59,60]. While expression of *MnSOD* in the nutrient addition gradient differed among genotypes, *GPX* was uniformly downregulated, even below the detection limit, at high nutrient addition and strongly upregulated (on average 44-fold, Figure 4e) at low nutrient addition in all genotypes. Interestingly, all genotypes challenged with the low level of nutrient addition showed the presence of oxidative stress via overexpression of *GPX* but not *MnSOD*. *GPX* was also found to be more strongly regulated than *MnSOD* in the stress response of *P. australis* to increased salinity, CO_2,_ and temperature by [59]. This may indicate that, for *P. australis*, the glutathione antioxidant system plays a greater role in acclimation than superoxide dismutase, and the change in its expression can be a useful indicator of oxidative stress. The presence of oxidative stress at low nutrient addition and its absence at high nutrient addition indicates that the plants encountered stressful conditions at low nutrient addition, which in this study was intended to represent medium nutrient availability in unfertilized fens in central Europe, and favorable conditions at high nutrient addition, which was in a range that may be encountered in treatment wetlands [47,49,50,51,59,60]. Therefore, the nutrient addition gradient seems suitable for detecting different plant strategies, while the water level gradient did not include stressful conditions for *P. australis*.

Along the nutrient addition gradient, we observed significant differences in the productivity of the genotypes under favorable conditions at very high nutrient addition, but they exhibited similar poor productivity under stressful conditions at low nutrient addition. Where significant differences occurred under stressful conditions at low nutrient addition (e.g., root biomass), the overall high productive genotype *Rue1* was still more productive than the overall low productive genotype *PV1*; genotype *PV1* did not seem to have an advantage under stressful conditions at low nutrient addition as the plant economics spectrum would suggest [40]. Ultimately, growth rate, a central consequence of different plant strategies and represented as shoot elongation rate in our study, did not reveal consistent differences between genotypes along the gradients.

Genotype *PV1* may be described as a ‘translocation type’ according to [38], investing in storage tissue (rhizome) with higher rhizome mass fraction and lower root mass fraction than all other genotypes over large parts of both gradients. In contrast, *Rue1* follows the ‘assimilation type’ investing in acquisition tissue (roots) with the highest mean root mass fraction and lowest mean rhizome mass fraction across the gradients. In line with the findings of Kühl et al. (1997) [38], our ‘translocation type’ *PV1* produced significantly less above- and belowground biomass than the ‘assimilation type’ *Rue1*. The attribution of the genotypes to these strategies is, however, not supported by the N content and the C/N-ratio of aboveground biomass at winter harvest [61], whereas this was a key distinguishing feature between strategy types by Kühl et al. (1997) [38].

Additionally, neither root nor leaf functional traits nor photosynthetic rate supported our hypothesis of different plant strategies among the genotypes. The mean photosynthetic rate across the gradients was higher in genotypes *Rue1*, *Rue2,* and *PV2* compared to *Ka* and *PV1*, which corresponds with the gene expression data, showing stronger regulation of major photosynthetic and transport genes for *Rue1* and *Rue2* than for *Ka* in changed conditions. While the mean photosynthetic rate showed a similar pattern to the productivity of the genotypes, there were only a few significant differences in photosynthetic rate between the genotypes along the environmental gradients. High phenotypic plasticity in photosynthetic rate has been reported for *P. australis*, and our results are in accordance with studies that report only minor differences in the photosynthetic rate of *P. australis* genotypes grown in a common environment [28,62]. Contrary to our hypothesis, the ‘fast’ type *Rue1* showed a comparatively low specific leaf area (SLA), while the ‘slow’ type *PV1* showed a comparatively high SLA. Regarding specific root length (SRL), differences between genotypes were inconsistent or non-existent. Both the high- and low-productive genotypes *Rue1* and *PV1* had a high mean SRL, while the medium-productive *Rue2* had the lowest mean SRL. Furthermore, *Rue1* had a higher leaf dry matter content (LDMC) than *PV1* across both gradients and a higher root dry matter content (RDMC) than *PV1* in the nutrient addition gradient. These results suggest that SLA was coordinated with tissue density but not with SRL and, more importantly, not with growth rate and productivity, within the studied genotypes. Similarly, Kramer-Walter et al. (2016) [63] found root tissue density to be coordinated with aboveground traits while SRL was orthogonal to the plant economics spectrum. Our finding of the absence of distinct plant strategies is supported by Wright & Sutton-Grier (2012) [64], who found only weak support for the leaf economics spectrum in 23 wetland species. Pan et al. (2019) [65] suggest that, while wetland plants can exhibit a leaf economic spectrum, these traits are decoupled from other wetland adaptive traits, such as root porosity, since these suits of traits are driven by different environmental mechanisms. In addition to evidence from these multi-species studies, Hu et al. (2015) [45] found coordination of leaf economic traits providing evidence for a within-species leaf economics spectrum of *P. australis*. However, the authors did not link the leaf traits to the growth rate or productivity of the *P. australis* genotypes. In our study, we considered both plant economic traits as well as growth rate and productivity, and although we found coordination of leaf economic traits, we could not link them to growth rate or productivity to form a sound plant economics spectrum.

### 3.3. Performance of P. australis Genotypes with Regard to Paludiculture Cannot Be Predicted Using the Plant Economics Spectrum

Differences in productivity between *P. australis* genotypes were evident only under favorable, not under stressful conditions, and functional traits did not contribute to forming a sound plant economics spectrum in our study along gradients of resource availability. Based on our data, the performance of *P. australis* genotypes cannot be predicted based on functional traits or the plant economics spectrum. Nevertheless, our study showed large differences in productivity and morphology between genotypes stemming from only a small subset of the species’ range. A targeted selection of suitable genotypes can therefore have a strong influence on the performance and profitability of a paludiculture. We recommend long-term genotype trials assessing key utilization criteria, similar to crop variety trials carried out in agriculture or provenance trials in forestry. Due to the large variability among *P. australis* genotypes, findings should not be generalized and are only valid for the tested genotypes [27]. Trials should therefore screen a large number of genotypes and explore local-to-regional differentiation. For *P. australis* in northeast Germany, we propose to include regional genotypes from existing thatching reed stands with desirable quality. Such trials should be carried out under different paludiculture scenarios, taking into account various water regimes and levels of nutrient availability typical for degraded peatlands. Finally, such trials need to run for at least three years, as newly established *P. australis* stands reach maturity for harvest two to three years after planting [66].

## 4. Materials and Methods

We grew five genotypes of *Phragmites australis* (Cav.) Steud. [67] in two mesocosm experiments and investigated their productivity, morphology, photosynthetic rate, functional traits, and gene expression along gradients of (A) nutrient addition and (B) water level.

### 4.1. Plant Material

Rhizomes of five *P. australis* genotypes were collected in thatching reed stands in northeast Germany, reflecting small-scale, regional variation within *P. australis*, in autumn 2018 (Table 1; genotyping according to [32]). Plants were grown from meristematic tissue using in-vitro propagation. From January to March 2019, plants were kept in a greenhouse and then acclimatized outside for two months.

### 4.2. Study Design

The experiments took place in Greifswald, Germany, from May 2019 to February 2020 and consisted of (A) a gradient of 14 nutrient addition levels and (B) a gradient of 15 water levels. Plants were grown in plastic tubes (h = 60 cm, d = 20 cm) filled with peat (*Sphagnum* peat, pH = 5.6–6.4, adjusted with carbonated chalk, unfertilized; Torfwerk Moorkultur Ramsloh, Saterland, Germany). Three clones per genotype were planted per tube.

Tubes in the nutrient addition gradient were sealed at the bottom and filled with 5 cm of expanded clay below the peat substrate. A flexible pipe running to the bottom of each tube connected them to the water reservoir of the respective treatment level (communal tap water, Table A1). The water level in the tubes was constantly held at the soil surface. Nutrient addition ranged from 50.2–3982.9 kg nitrogen (N) ha^−1^ yr^−1^ increasing by a factor of 1.4 over the treatment levels (Table A2). These nutrient addition values cover a range from medium nutrient availability in unfertilized fens in central Europe [47] to values that do not typically occur in near-natural fens but may occur in constructed wetlands for treatment of sewage or field runoff [49,50,51]. Plants were fertilized with N, phosphorus (P) and potassium (K) in the form of NH_4_NO_3_, (NH_4_)_2_HPO_4_ and K_2_CO_3_ dissolved in demineralized water (N/P = 5.12, N/K = 0.51). The fertilizer was applied dissolved in 0.5 L water at seven dates throughout the growing season at biweekly intervals, with one-third of the total amount of nutrients given at the first date (11 June 2019) and the remaining amount divided equally among the other dates.

Tubes in the water level gradient were closed at the bottom with two layers of water-permeable root fleece (polypropylene, 150 g m^−2^) and placed inside 1000 L containers (1 × 1 × 1 m) filled with communal tap water. Two water-level treatments were realized per container by placing the tubes on wooden platforms of different heights. The water level gradient ranged from 40 cm above the soil surface to 45 cm below the soil surface (Table A3). To avoid complete flooding of plants, water levels of 10–40 cm above the soil surface were raised gradually with plant growth to the desired height, which was reached latest at the beginning of August. Coarse nets were placed on the soil surface of tubes in the water level gradient to prevent peat from floating under flooded conditions. Plants were fertilized following the same procedure and schedule as in level 8 of the nutrient addition gradient. The nutrient solution was poured either directly into each tube (water level at or below the soil surface) or into the water above each tube (water level above the soil surface).

The mesocosms experienced ambient weather conditions. During the experiment, temperatures ranged from −2 °C (1 November 2019) to 37 °C (30 June 2019), the coldest month being December (mean minimum temperature 2.45 °C, mean maximum temperature 5.68 °C) and the warmest being June (15.53 °C and 25.57 °C, respectively). The lowest monthly precipitation was 29 mm in July, and the highest was 96 mm in November.

### 4.3. Growth, Morphology, and Biomass

Maximum plant height and the number of stems per tube were measured weekly from 13 June 2019 to 18 September 2019. Shoot elongation rate [ $\mathrm{cm}\;\times {\;\mathrm{day}}^{-1}$ ] was calculated from the maximum plant height of subsequent measurements. Biomass (per tube) was harvested in February 2020 and separated into above (leaves, stems)- and belowground (roots, rhizomes) biomass. Stems were separated into two random subsamples, of which one was used for further analyses. The fresh weight of both subsamples was recorded. Leaves and one stem subsample were dried for 72 h at 60 °C. Water content from the fresh and dry weight of this subsample was used to calculate the dry weight of the second subsample. The dry weight of leaves and both stem subsamples were summed up to the total aboveground dry weight. Belowground biomass was washed free of substrate and dried at 60 °C to constant weight. Samples with a high amount of biomass (*n* = 110) were divided into two parts, weighed, and only one part washed. Root and rhizome dry weight were extrapolated to the total weight. Biomass allocation was calculated as belowground mass fraction, root mass fraction, and rhizome mass fraction, all relative to total plant biomass. A theoretical yield for assumed paludiculture conditions (60 to 180 kg N ha^−1^ yr^−1^ nutrient addition [47] and 0 to +30 cm water level [48]) was calculated as the mean of fitted values of aboveground biomass in the respective ranges.

### 4.4. Photosynthetic Rate and Functional Leaf and Root Traits

The photosynthetic rate was measured by leaf gas exchange using the LCi-SD Leaf Chamber Analysis System (ADC BioScienfitic Limited, Hoddesdon, Herts, England) with external PLU5 LED light unit (given PAR, PAR Q_leaf_ (g) = 1500 µmol m^−2^ s^−1^, PAR Q_given_ = 1630 µmol m^−2^ s^−1^) fitted with the narrow leaf chamber without a radiation shield (area: 5.80 cm^2^, Hfac: 0.168, rb: 0.30, Tl_mtd_: measured, Trw = 0.92, u_set_ = 200 µmol s^−1^). Only leaves big enough to fill the chamber were used. Measurements were carried out from 13 August 2019 to 3 September 2019 in four campaigns of two days each in the nutrient- and water-level gradient, respectively. A blocked rotating sampling design ensured that each plant was measured at least once at different times (morning, noon, and afternoon) to avoid the influence of diurnal changes on the photosynthetic rate. Measurements were taken at the second fully expanded leaf from the top. If another leaf was measured, the photosynthetic rate was corrected for leaf position to resemble the photosynthetic rate at leaf position two. The correction was based on a LOESS spline (span = 1) of photosynthetic rate versus leaf position of all leaves of 30 plants in different treatment levels.

Specific leaf area (SLA) was measured for vital leaves from 9–11 October 2019. The leaf blade of the third fully expanded leaf of the three highest stems per tube was collected, kept in sealed plastic bags with a moist paper towel at 4 °C for at least 7 h, dabbed dry, weighed fresh at 0.1 mg precision, and scanned with a flatbed scanner (400 dpi, Epson Perfection V800 Photo, EPSON Deutschland GmbH, Meerbusch, Germany). Leaves were dried at 60 °C for 72 h before recording dry weight. Leaf area was measured using ImageJ (Version 1.52a; [68]). SLA was calculated as the one-sided area of a fresh leaf divided by its dry weight and leaf dry matter content (LDMC) as dry weight divided by the fresh weight of a leaf [69].

Specific root length (SRL) was measured for roots from the upper 15 cm of soil sampled from 2–3 December 2019. Roots were stored in sealed plastic bags at 4 °C, then carefully washed free of adherent substrate. The roots were scanned in a transparent flat container arranged to overlap as little as possible in a minimum amount of water (flatbed scanner with transmitted light, Epson Perfection V800 Photo, EPSON Deutschland GmbH, Meerbusch, Germany; 600 dpi; 8-bit greyscale picture). They were then dabbed dry, and their fresh and dry (48 h, 60 °C) weight was recorded at 0.1 mg precision. Root length with Kimura root length correction [70] was measured with the IJRhizo macro [71] for ImageJ (Version 1.53k; [68]) (600 dpi, width of excluded border 0 pixels, size of the smallest measured particle 0.5 mm^2^, circularity 0.75, user-defined lower threshold value 0, upper threshold value 220 and “Perform particle cleaning”, “Perform root length correction” and “Perform Kimura root length correction” activated). SRL was calculated as the ratio of root length to dry weight of each sample [69] and root dry matter content (RDMC) as dry weight divided by the fresh weight of each sample. IJRhizo also supplied a measure of average root diameter (Mean_Dia derived from corrected root length RLc and projected root surface area Surf_Area).

Aerenchyma volume was assessed as root porosity of fine roots (diameter < 2 mm, first and second order only) from the upper 10 cm of the soil horizon sampled on 25 October 2019. Root porosity was analyzed with the pycnometer method according to [72] using distilled and degassed water of 28 °C. First, the water-filled pycnometer (volume 100.71 cm^3^) was weighed at 0.1 mg precision ( PW ). About 1 g of fresh root biomass was dabbed dry, weighed ( FW ), placed in the pycnometer, and the pycnometer with submerged roots was weighed again after removing excess water ( W ). Then, it was placed into an exsiccator (Rotilabo^®^-Exsikkator Modell 3, Carl Roth GmbH+Co. KG, Karlsruhe, Germany) to replace the air in the roots with water by vacuum infiltration. A negative pressure of 0.3–0.4 bar was applied for five minutes, then relieved shortly and reapplied three to four times again until no more air bubbles ascended from the roots. After stirring with a wooden stick to cause all remaining air bubbles to rise, the pycnometer was topped up with water and weighed ( $W_{\inf}$ ). Root porosity was quantified according to Formula (1) [72]:

(1) $$\mathrm{root}\;\mathrm{porosity}\;\left[\%\right]=\frac{100\times \left(W_{\inf\;}\left[g\right]-\;W\;\left[g\right]\right)}{\left(\mathrm{PW}\;\left[g\right]+\mathrm{FW}\;\left[g\right]-\;W\left[g\right]\right)}$$

### 4.5. Gene Expression Analysis

A total of 18 samples from genotypes *Rue1*, *Rue2,* and *Ka* from 3 water levels (−45, 0, 40 cm) and 3 nutrient addition levels (50.2, 377.9, 3982.9 kg N ha^−1^ yr^−1^) were taken on 2 October 2019. Per sample, tissue of 3 leaves was collected from 3 randomly chosen stems; a 0.5 cm^2^ piece of a fully developed and visually healthy leaf was taken in the middle position from the leaf base. The material was stored in RNAlater solution (Thermo Scientific, Waltham, MA, USA) at −20 °C. Leaf material from one sample was pooled, and total RNA was extracted using the innuPrep Plant RNA Kit (Analytik Jena, Jena, Germany) according to the manufacturer’s protocol. The purity and integrity of RNA extracts were measured by NanoDrop Lite spectrophotometer (Thermo Scientific, USA) and via electrophoresis on 1.5% (*w*/*v*) agarose gel. Subsequently, 1 mg RNA was treated with 1U DNase and synthesized into cDNA using the First Strand cDNA Synthesis Kit (Thermo Scientific, USA) with the random hexamer primers.

Gene expression of six genes of interest (GOI) was analyzed using three reference genes (REF) (Table A4). The GOI included three photosynthetic genes (*RbcS*, Ribulose bisphosphate carboxylase small chain; *PGK,* Phosphoglycerate kinase; *PRK*, Phosphoribulokinase), two oxidative stress response genes (*GPX*, Glutathione peroxidase; *MnSOD*, Manganese superoxide dismutase), and one transporter gene (*NHA*, Na^+^/H^+^ antiporter). As REF, *EF1α* (Elongation factor 1α*)*, *PP2A4* (Serine/threonine protein phosphatase catalytic subunit 4), and *UBC* (Ubiquitine conjugating protein) were used.

To design primers for *EF1α* and *PP2A4*, sequences from different Poaceae species were retrieved from GenBank (AP014959.1, FP098428.1, XM_003577268.4, AK354224.1, XM_020318228.1, XM_025951679.1, XM_004962211.3, EF581011.1, XM_021449142.1, AK455966.1 and NC_029265.1, NW_017932705.1, MG461318.1, XM_025937507.1, respectively) and aligned using MEGA X [73]. To test that primers do not amplify DNA, PCR with two RNA samples after DNase treatment was performed with primers for each GOI and REF. A cDNA sample was used as a positive control. The absence of amplicons was proven by agarose gel electrophoresis.

PCR products obtained for all the primer pairs used in this study were sequenced and tested for specificity via BLAST; sequences for genes *PP2A4* and *EF1α* were uploaded to GenBank (Accession numbers: OQ376569 and OQ376571, respectively).

The gene maximization set-up was applied (on one plate, all genes were analyzed together for three samples with the same genotype and 3 treatment levels). Three technical replicates were used; no template control for each primer pair was included. All qPCR reactions were performed with one protocol. Each 10 µL reaction mixture contained 5 µL PowerUp SYBR Green Master Mix (Applied Biosystems, Waltham, MA, USA), 0.1 µM of each primer, 2 µL nuclease-free water, and 1 µL cDNA (diluted 1:2). The qPCR was conducted with a 7500 Fast PCR System (Applied Biosystems, USA) on the ‘Fast Real Time PCR’ setting and the following program: (i) 2 min at 50 °C; (ii) 45 cycles consisting of 15 s at 95 °C, 15 s at 56 °C and 30 s at 72 °C; (iii) 1 min 72 °C; (iv) a melting curve step (heating from 60 to 95 °C with a rate of 0.1 °C per second with continuous measurement of fluorescence).

Determination of quantitative cycles (Cq) was performed using the automatic calculation of the 7500 Software 2.0.6 (Applied Biosystems). A standard curve was built for each pair of primers, and PCR efficiency *E* (%) was calculated.

### 4.6. Data Analysis

All statistical tests and data visualization were performed in R 3.6.0 [74] using the package ggplot2 3.3.0 [75] and RStudio IDE 1.2.5003 [76].

This study aimed at observing potentially non-linear patterns of response variables along environmental gradients. A powerful tool to answer this kind of question are gradient experiments which maximize treatment levels by minimizing replication [77]. To unravel these response patterns, a graphical analysis was performed. Each response parameter was plotted over the environmental gradient and smoothed conditional means were calculated by Local Polynomial Regression Fitting using the ‘loess’ function implemented in R. Span was adjusted to produce smooth curves without multiple local extrema based on the assumption that multiple maxima are unlikely in an autecological setting without competition. Confidence intervals (CI) displayed around the conditional means were used to assess the significance of effects at a level of α = 0.05. The environmental gradient was considered to have a significant effect if a straight horizontal line could not be fitted inside the 95% CI [78]. Two genotypes were considered significantly different if their 83% CIs did not overlap [79,80].

For the gene expression analysis, the stability of reference genes was determined by refFinder [81] and showed that *UBC < PP2A4* < *EF1α*. *UBC* was excluded from the analysis due to unstable expression. PCR efficiency (*E)* varied from 92 to 163% (Table A4). Normalized relative quantities (NRQ), which show the fold of change in gene expression compared to the control sample, were calculated according to [82]. As a control sample, a sample with a medium treatment level was used (0 cm and 27.1 kg N ha^−1^ yr^−1^ for water and nutrient addition levels, respectively). Before statistical analyses, NRQ values of three biological replicates were standardized by log transformation, mean centering, and autoscaling [83]. To detect significant differences in NRQ among the two treatment levels, Student t-tests with Benjamini-Hochberg correction were performed. T-test assumptions were tested via Levene’s test for variance homogeneity and the Kolmogorov-Smirnov test for normal data distribution.

## 5. Conclusions

This study demonstrates the high variability among *P. australis* genotypes, which has considerable implications for paludiculture. Under likely paludiculture conditions, the five studied genotypes differed up to twofold in aboveground yield for possible further utilization and up to 1.7-fold in belowground biomass productivity, suggesting differences in their peat-forming potential. Furthermore, the genotypes exhibited a distinct morphology (height, growing density) relative to each other. Productivity and morphology are likely retained when transplanting, so careful selection of *P. australis* genotypes for paludiculture is recommended to ensure success and profitability. While the nutrient addition gradient offered a range of stressful to favorable conditions, the genotypes did not exhibit different strategies according to the plant economics spectrum based on their functional traits and their performance along resource gradients. Based on these results, we conclude that the plant economics spectrum cannot be used to predict the suitability of *P. australis* genotypes for paludiculture. Instead, large-scale and long-term genotype trials will be necessary to select suitable *P. australis* genotypes for paludiculture.

## Figures and Tables

**Table 1:** Origin of the five *P. australis* genotypes used in this study; meristematic tissue was collected in these thatching reed stands and used for in vitro propagation.

Genotype	Area of Origin	Approximate Collection Coordinates	Haplotype (*TrnT-TrnL*)
*Rue1*	Lieschow peninsula, Rügen	54.43480° N, 13.18848° E	T4b
*Rue2*	Lieschow peninsula, Rügen	54.43574° N, 13.19760° E	T4b
*PV1*	Lower Peene valley	53.85401° N, 13.78094° E	T4c
*PV2*	Lower Peene valley	53.85589° N, 13.79369° E	T4b
*Ka*	Karrendorfer Wiesen near Greifswald	54.15396° N, 13.38301° E	T7c

## Data Availability

The data used in this study for direct analysis as well as gene expression data and calculations are publicly available via FigShare [https://doi.org/10.6084/m9.figshare.21856734, accessed on 8 February 2023]. All sequence data are available from GenBank via the Accession Numbers given in the main text (https://www.ncbi.nlm.nih.gov/genbank/ (accessed on 8 February 2023)).

## Supplementary Materials

The following supporting information can be downloaded at: https://www.mdpi.com/article/10.3390/plants12051045/s1; Figure S1: Root and rhizome biomass dry weight of five *P. australis* genotypes (*Rue1*, *Rue2*, *PV1*, *PV2*, *Ka*; Table 1)., Figure S2: Shoot elongation rate of five *P. australis* genotypes (*Rue1*, *Rue2*, *PV1*, *PV2*, *Ka*; Table 1).; Figure S3: Biomass allocation of five *P. australis* genotypes (*Rue1*, *Rue2*, *PV1*, *PV2*, *Ka*; Table 1).; Figure S4: Photosynthetic rate of five *P. australis* genotypes (*Rue1*, *Rue2*, *PV1*, *PV2*, *Ka*; Table 1).; Figure S5: Root porosity of five *P. australis* genotypes (*Rue1*, *Rue2*, *PV1*, *PV2*, *Ka*; Table 1).; Figure S6: Functional leaf traits of five *P. australis* genotypes (*Rue1*, *Rue2*, *PV1*, *PV2*, *Ka*; Table 1).; Figure S7: Functional root traits of five *P. australis* genotypes (*Rue1*, *Rue2*, *PV1*, *PV2*, *Ka*; Table 1).

## Appendix A

**Table A1:** Analysis of communal tap water used for irrigation of the mesocosm experiment. Tap water was a mixture of water from waterworks Schönwalde (60%) and Hohenmühl (40%). Values in this table are calculated accordingly from data of [84].

Parameter	Unit	Method	Value
pH		DIN EN ISO 10523	7.39
NH_4_^+^	[mg L^−1^]	DIN EN ISO 11732	0.014
NO_2_^−^	[mg L^−1^]	DIN EN ISO 13395	0.016
NO_3_^2−^	[mg L^−1^]	DIN EN ISO 10304-1	1.72
PO_4_^3−^	[mg L^−1^]	DIN EN ISO 15681-1	0.038
K^+^	[mg L^−1^]	DIN EN ISO 11885	2.84

**Table A2:** Fertilizer in the nutrient addition gradient. Target amount of nitrogen (N) [kg ha^−1^ yr^−1^] and resulting amount of chemicals (NH_4_)_2_HPO_4_, NH_4_NO_3_ and K_2_CO_3_ that were dissolved in 0.5 L distilled water and used for fertilization per pot [g yr^−1^] based on a pot surface area of 314.16 cm^2^, an N/P-ratio of 5.12 and an N/K-ratio of 0.51.

Level	Target Amount of N [kg ha^−1^ yr^−1^]	Total Amount of Fertilizer per Pot [g yr^−1^]		
		(NH_4_)_2_HPO_4_	NH_4_NO_3_	K_2_CO_3_
1	50.2	0.131	0.371	0.545
2	70.2	0.184	0.519	0.763
3	98.3	0.257	0.727	1.069
4	137.7	0.360	1.018	1.496
5	192.8	0.504	1.425	2.094
6	269.9	0.706	1.995	2.932
7	377.9	0.988	2.793	4.105
8	529.0	1.383	3.910	5.747
9	740.6	1.936	5.474	8.046
10	1036.8	2.710	7.664	11.264
11	1451.5	3.795	10.729	15.770
12	2032.1	5.312	15.021	22.078
13	2844.9	7.437	21.029	30.909
14	3982.9	10.412	29.441	43.273

**Table A3:** Water level in relation to soil surface in the water level gradient. Positive numbers are water levels above soil surface, negative numbers are water levels below soil surface.

Level	Water Level [cm]
1	−45
2	−38
3	−31
4	−24
5	−18
6	−12
7	−6
8	0
9	+5
10	+10
11	+16
12	+22
13	+28
14	+34
15	+40

**Table A4:** Primers used for the gene expression analysis of *Phragmites australis* at different water level or nutrient addition treatments.

Gene	Protein	Primer	PCR Efficiency [%]	Product Size [bp]	Primer Sequence (5’–3’)
*RbcS*	Ribulose bisphosphate carboxylase small chain	rbcS-fw ^†^ rbcS-rev ^†^	107.5	150	CAG GTG CAT GCA GGT GTG G CCG ACC TTG CTG AAC TCG AGG
*PGK*	Phosphoglycerate kinase	Phgly-fwd ^†^ Phgly-rev ^†^	120.5	149	GTT TGC TGT AGG AAC TGA GGC TGT CAC CTC CCG TTG AAA TGT GGC TCA
*PRK*	Phosphoribulokinase	Phori-fwd ^†^ Phori-rev ^†^	92.0	183	GAC TCT TAC TTC GGC CAT GAG GTA TCA GAA GAG ACC TGT TCC ATT GTT GCT
*GPX*	Glutathione peroxidase	GPX-fwd ^†^ GPX-rev ^†^	NA ^‡^	163	GAA TTC CCT ATT TTT GAC AAG GTT GA GCG CAT AGC GAT CCA CAA C
*MnSOD*	Manganese superoxide dismutase	SOD-fwd ^†^ SOD-rev ^†^	145.7	147	CAA GGA TCT GGA TGG GTG TGG C GTA GTA CGC ATG CTC CCA GAC AT
*NHA*	Na+/H+ antiporter	NaH-fwd ^†^ NaH-rev ^†^	118.0	170	GTG CGG CTT TTG AAT GGT GTG GGG AAC TGG ACA CTG GAC TGT AAA
*EF1α*	Elongation factor 1*α*	EF1a-fwd EF1a-rev	107.2	109	TGA GGC TGG TAT CTC CAA GGA AGT GGT GGC RTC CAT CTT GTT GC
*PP2A4*	Serine/threonine protein phosphatise 4 catalytic subunit-like	PP2A4-fwd PP2A4-rev	110.6	138	GTG TGC GTA GCT TRG ATC GTG TCC GAT ATG TCC TGY CCA AAA GTG TAG CCA G
*UBC*	Ubiquitine conjugating protein	UBC-fwd ^†^ UBC-rev ^†^	113.1	117	CTT CAA GCC RCC AAA GGT MTC GAT ATT GTC AAA GCA GGG CTC CA

^†^ [54], ^‡^ not determined due to the absence of a signal at the highest dilutions.
