# Correction: Haldan et al. Choose Wisely: Great Variation Among Genotypes of Promising Paludiculture Crop *Phragmites australis*. *Plants* 2023, *12*, 1045

**DOI:** 10.3390/plants15182812

**Published:** 2026-09-14

**Authors:** Kerstin Haldan, Kristina Kuprina, Meike Ingeborg Haase, Fabian Kieckhäfer, Lisa Schade, Joraine Schmoldt, Lina Stella Schock, Marthe Stein, Alexander Wille, Martin Schnittler, Manuela Bog, Jürgen Kreyling

**Affiliations:** Institute of Botany and Landscape Ecology, University of Greifswald, Partner in the Greifswald Mire Centre, 17489 Greifswald, Germany

**Calculation error in the nutrient treatment**

When calculating the amount of fertilizer needed to reach the target amounts of N, P and K in the experiment, a calculation mistake was made in the original publication [1]. This means that the fertilizer amounts which we added to the experiment resulted in different nutrient target amounts than stated in the original paper. The actual target amount of N addition is 13.94 times higher than stated in the paper. The actual nutrient addition gradient ranges from addition of 50.2 to 3982.9 kg N ha^−1^ yr^−1^. In the water table treatments, plants were fertilized with a nutrient solution equivalent to the middle level (level 8) of the nutrient addition treatment. This nutrient addition was erroneously stated as 37.9 kg N ha^−1^ yr^−1^ in the original paper, but it actually amounts to 529.0 kg N ha^−1^ yr^−1^. Samples for gene expression analysis were taken from three nutrient addition levels (50.2, 377.9, 3982.9 kg N ha^−1^ yr^−1^). The target amount of N as well as the amounts of chemicals used for fertilization were specified in Table A2. The amounts of chemicals used for fertilization presented in Table A2 are correct; however, the specified target amount of N is not. Therefore, we have attached a corrected version of Table A2 to this correction. As the nutrient addition was different than originally intended, nutrient ratios were also different than intended. The N/P-ratio in our experiment was 5.12, the N/K-ratio was 0.51.

For the reader, this essentially means that all amounts of nutrient addition in the original paper, given as kg N ha^−1^ yr^−1^, have to be multiplied by 13.94 to obtain the actual amounts of N addition. Special attention must be paid whenever the nutrient additions of our experiment are mentioned in the results and discussion. All patterns of the originally presented figures remain, but the x-axis of the nutrient addition gradient changes as shown in the corrected versions of all figures attached to this correction.

Under assumed paludiculture nutrient conditions (nutrient availability in unfertilized wet meadows and fens in western Europe, 60–180 kg N ha^−1^ yr^−1^ [2]), the investigated genotypes did not differ significantly regarding their productivity and differed less than described in the original paper regarding their morphology, especially their stem number. Differences in peat formation potential based on belowground productivity were not significant between genotypes under assumed paludiculture nutrient conditions.

Theoretical above- and belowground yields under assumed paludiculture conditions were calculated based on the genotypes’ productivity in the range of nutrient addition of 60 to 180 kg N ha^−1^ yr^−1^ and therefore have to be corrected for the corrected nutrient addition. Theoretical aboveground yield under assumed paludiculture conditions was actually between 6.20 t ha^−1^ (genotype PV1) and 9.95 t ha^−1^ (genotype Rue1) in the nutrient addition gradient. The most (Rue1) and least productive (PV1) genotypes thus differed only 1.6-fold in theoretical aboveground yield in the nutrient addition gradient. The same genotypes differed twofold in their theoretical aboveground yield in the water level gradient under assumed paludiculture conditions of 0 to +30 cm water level.

Theoretical belowground yield under assumed paludiculture conditions was actually between 16.39 t ha^−1^ (genotype PV1) and 23.23 t ha^−1^ (genotype Ka) in the nutrient addition gradient. The most (Ka) and least productive (PV1) genotypes differed only 1.4-fold in theoretical belowground yield in the nutrient addition gradient. Under assumed paludiculture conditions in the water level gradient (0 to +30 cm water level), the most productive genotype (Rue2, 34.37 t ha^−1^) and least productive genotype (PV1, 20.77 t ha^−1^) differed 1.7-fold. Notably, the most productive genotypes regarding belowground yield under assumed paludiculture conditions were Ka in the nutrient addition gradient and Rue2 in the water level gradient, and not the aboveground most productive genotype Rue1.

Along the nutrient addition gradient, significant differences between genotypes in above- and belowground biomass occurred upwards of a nutrient addition of approximately 500 kg N ha^−1^ yr^−1^. Although our results now show that the genotypes investigated in our study did not clearly differ in their productivity under nutrient conditions typical for western European fens, the genotypes differed clearly when subjected to extreme nutrient conditions.

The nutrient addition values cover a range from medium nutrient availability in unfertilized fens in central Europe [2] to values that do not typically occur in near-natural, unfertilized fens, but may occur in constructed wetlands for treatment of sewage or field runoff [3,4,5]. Some of the *Phragmites australis* genotypes investigated in our study did not only survive, but even thrived under very high nutrient inputs. It is astounding that the plants experienced oxidative stress at low nutrient addition, which in this study was intended to represent medium nutrient availability in unfertilized fens in central Europe, but not at very high nutrient addition, which was in a range that may be encountered in treatment wetlands, as gene expression analysis in our study showed (Figure 4e). The differentiation of genotypes only at high nutrient addition highlights the value of gradient experiments which provide the ability to explore large gradients going beyond naturally occurring conditions to better understand the study species and gain insights which would otherwise be undiscovered.

Our paper still provides conclusive data and fills a research gap by revealing the performance of five *Phragmites australis* genotypes in a nutrient addition and a water level gradient. It now turned out to explore *Phragmites australis* performance at nutrient loads at and beyond natural conditions in fens in western Europe.

**Error in Table A2 and Table A2 Legend**

In the original publication, there was a mistake in Table A2 and in the legend for Table A2 as published. Due to the calculation error in the nutrient treatment described above, in the column “Target amount of N” wrong values were reported. In the Table legend, wrong values for the N/P-ratio and N/K-ratio were reported.

**Table A2:** Fertilizer in the nutrient addition gradient. Target amount of nitrogen (N) [kg ha^−1^ yr^−1^] and resulting amount of chemicals (NH_4_)_2_HPO_4_, NH_4_NO_3_ and K_2_CO_3_ that were dissolved in 0.5 L distilled water and used for fertilization per pot [g yr^−1^] based on a pot surface area of 314.16 cm^2^, an N/P-ratio of 5.12 and an N/K-ratio of 0.51.

Level	Target Amount of N [kg ha^−1^ yr^−1^]	Total Amount of Fertilizer per Pot [g yr^−1^]		
		(NH_4_)_2_HPO_4_	NH_4_NO_3_	K_2_CO_3_
1	50.2	0.131	0.371	0.545
2	70.2	0.184	0.519	0.763
3	98.3	0.257	0.727	1.069
4	137.7	0.360	1.018	1.496
5	192.8	0.504	1.425	2.094
6	269.9	0.706	1.995	2.932
7	377.9	0.988	2.793	4.105
8	529.0	1.383	3.910	5.747
9	740.6	1.936	5.474	8.046
10	1036.8	2.710	7.664	11.264
11	1451.5	3.795	10.729	15.770
12	2032.1	5.312	15.021	22.078
13	2844.9	7.437	21.029	30.909
14	3982.9	10.412	29.441	43.273

**Error in Figures 1, 4, S1 and S3–S7**

In the original publication, there was a mistake in Figures 1, 4, S1 and S3–S7. The x-axis labels showed wrong nutrient addition values due to the calculation error in the nutrient treatment described above. The corrected figures including their figure legends appear below.

**Figure 1:**
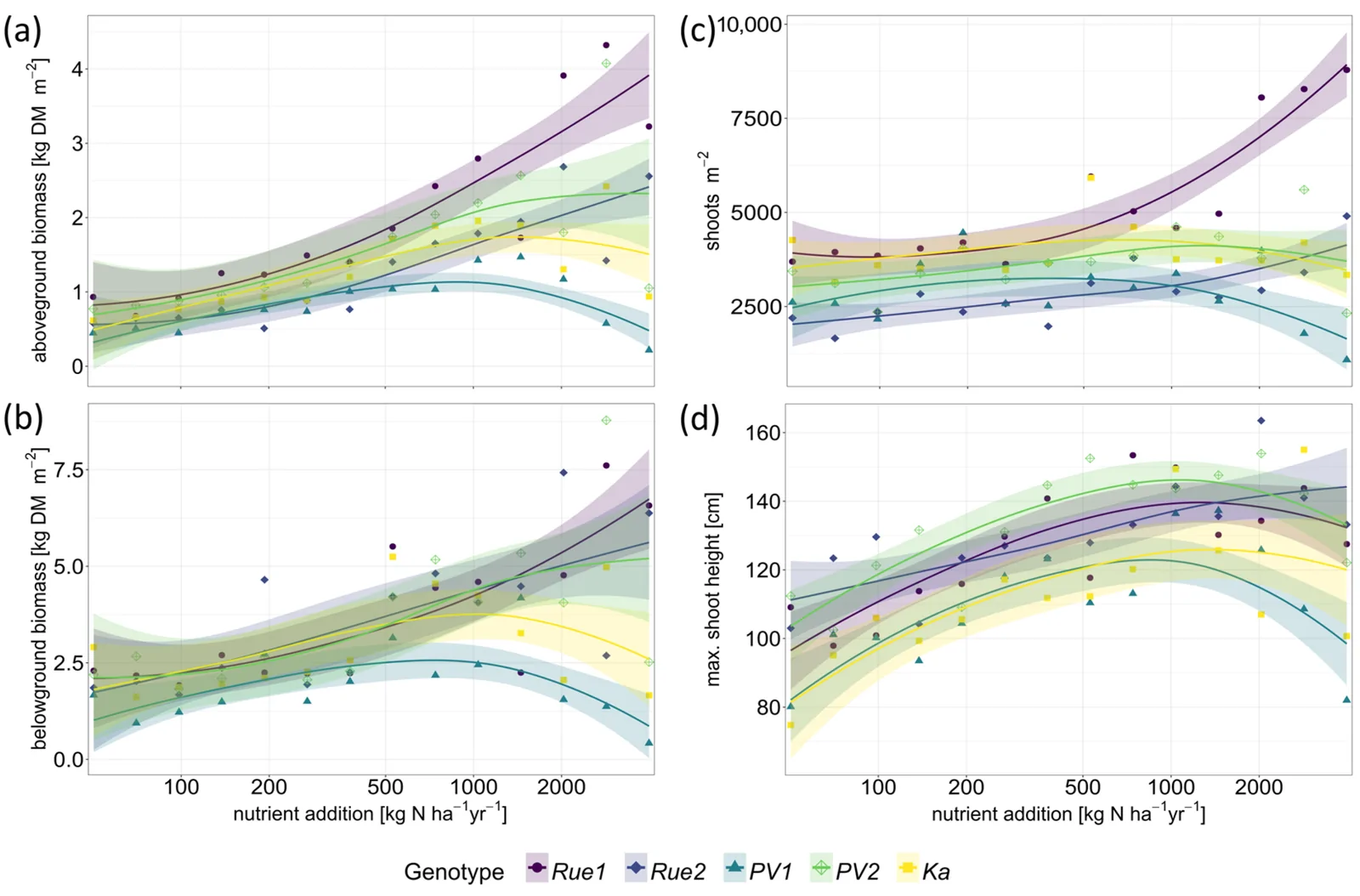
Biomass and morphology of five *P. australis* genotypes (*Rue1*, *Rue2*, *PV1*, *PV2*, *Ka*; Table 1) along the nutrient addition gradient. (**a**) Aboveground biomass dry weight [kg m^−2^] (span = 1.5) and (**b**) belowground biomass dry weight [kg m^−2^] (span = 1.5) at harvest in February 2020. (**c**) Number of shoots per m^2^ (span = 1.5) and (**d**) maximum shoot height [cm] (span = 1.8) at the end of the growing season on 18 September 2019. Symbols show original data points; lines are the smoothed local polynomial regression fittings (loess). Shaded areas around lines indicate 83% confidence intervals.

**Figure 4:**
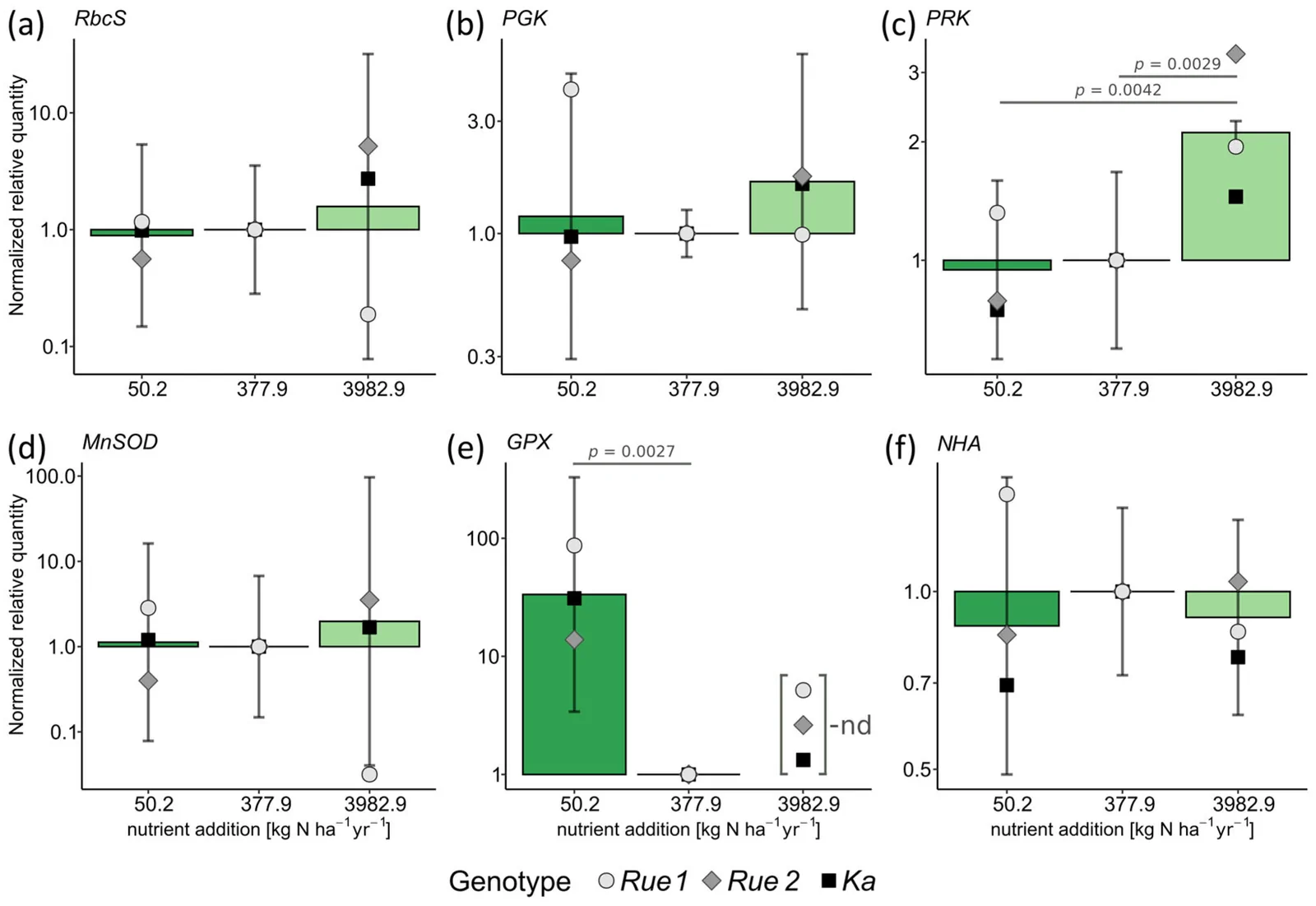
Normalized relative quantity of three photosynthetic genes ((**a**) *RbcS*, Ribulose bisphosphate carboxylase small chain, (**b**) *PGK*, Phosphoglycerate kinase, (**c**) *PRK*, Phosphoribulokinase), two oxidative stress response genes ((**d**) *MnSOD*, Manganese superoxide dismutase, (**e**) *GPX*, Glutathione peroxidase), and one transporter gene ((**f**) *NHA*, Na^+^/H^+^ antiporter) of three genotypes of *P. australis* (*Rue1*, *Rue2*, *Ka*; Table 1) at three levels of nutrient addition. Bar plots and error bars are built for the standardized data. Error bars show 95% confidence intervals, *p*-values (BH corrected) are given for pairs with statistically significant *t*-test results, nd—not determined (low expression).

**Figure S1:**
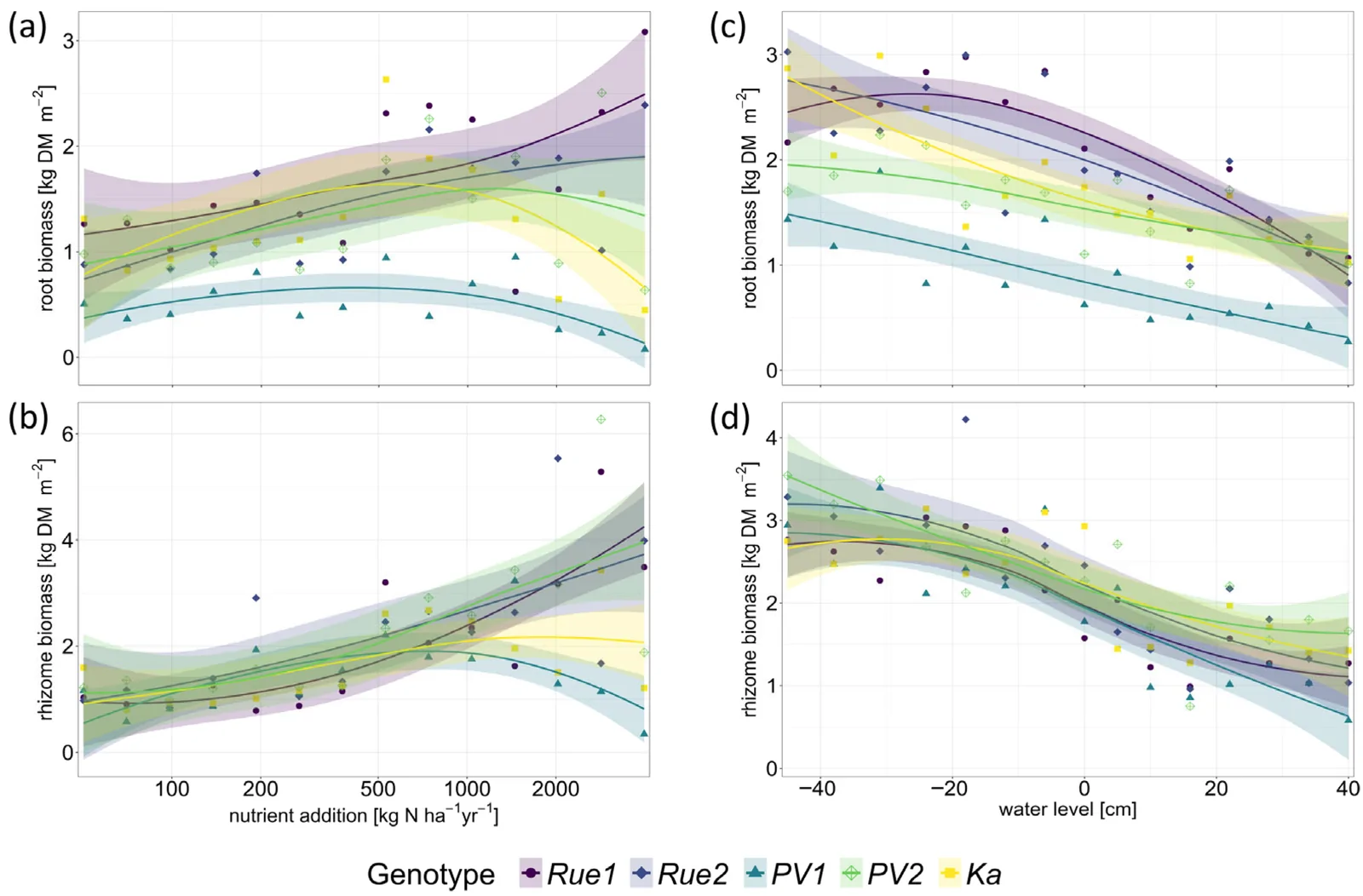
Root and rhizome biomass dry weight of five *P. australis* genotypes (Rue1, Rue2, PV1, PV2, Ka; Table 1). (**a**) Root biomass dry weight [kg m^−2^] (span = 1.8) and (**b**) rhizome biomass dry weight [kg m^−2^] (span = 2.0) along the nutrient addition gradient. (**c**) Root biomass dry weight [kg m^−2^] (span = 2.0) and (**d**) rhizome biomass dry weight [kg m^−2^] (span = 1.2) along the water level gradient. Symbols show original data points; lines are the smoothed local polynomial regression fittings (loess). Shaded areas around lines indicate 83% confidence intervals. Along the water level gradient, negative numbers represent water levels below ground and positive numbers represent water levels above ground.

**Figure S3:**
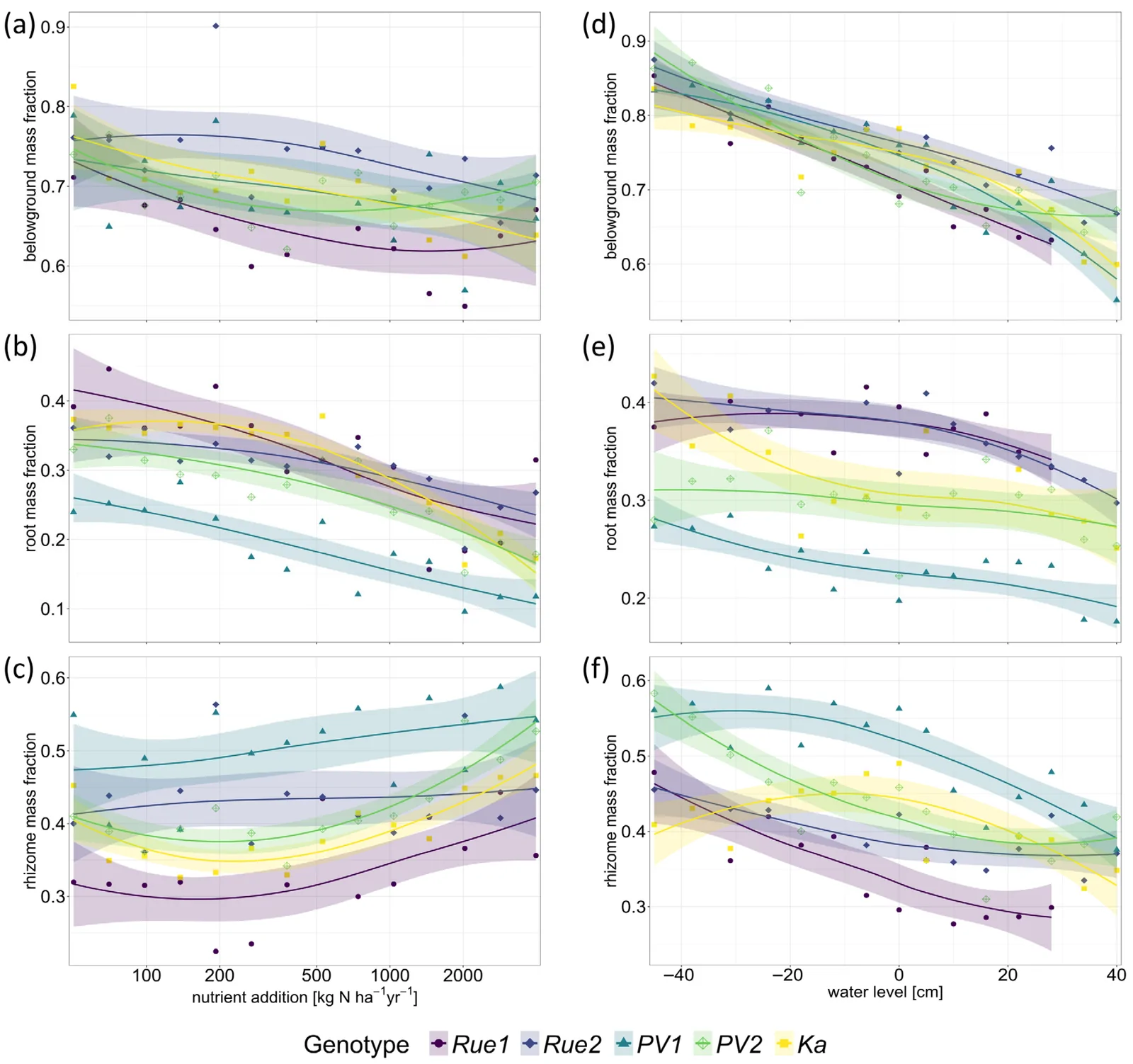
Biomass allocation of five *P. australis* genotypes (Rue1, Rue2, PV1, PV2, Ka; Table 1). (**a**) Belowground mass fraction (span = 1.8), (**b**) root mass fraction (span = 2.0) and (**c**) rhizome mass fraction (span = 1.8) along the nutrient addition gradient. (**d**) Belowground mass fraction (span = 1.2), (**e**) root mass fraction (span = 1.4) and (**f**) rhizome mass fraction (span = 1.2) along the water level gradient. Symbols show original data points; lines are the smoothed local polynomial regression fittings (loess). Shaded areas around lines indicate 83% confidence intervals. Along the water level gradient, negative numbers represent water levels below ground and positive numbers represent water levels above ground.

**Figure S4:**
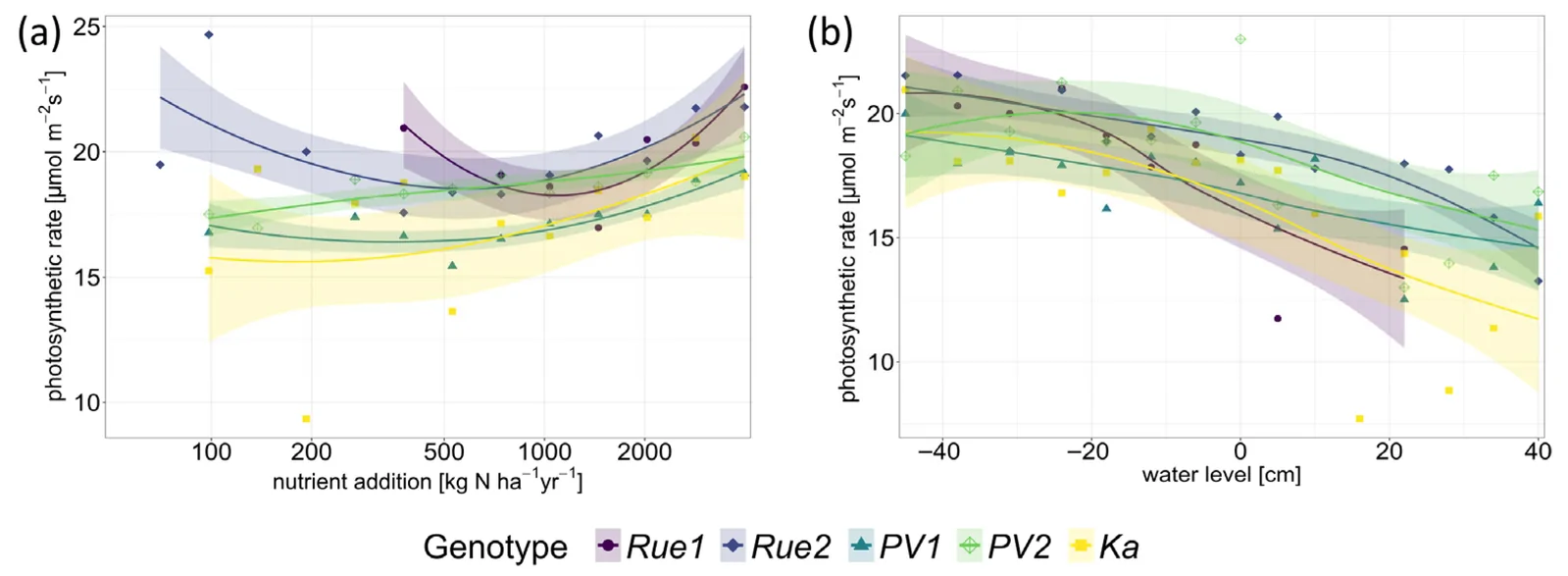
Photosynthetic rate of five *P. australis* genotypes (Rue1, Rue2, PV1, PV2, Ka; Table 1). (**a**) Photosynthetic rate [µmol m^−2^ s^−1^] along the nutrient addition gradient (span = 2.0). (**b**) Photosynthetic rate [µmol m^−2^ s^−1^] along the water level gradient (span = 1.5). Symbols show original data points; lines are the smoothed local polynomial regression fittings (loess). Shaded areas around lines indicate 83% confidence intervals. Along the water level gradient, negative numbers represent water levels below ground and positive numbers represent water levels above ground.

**Figure S5:**
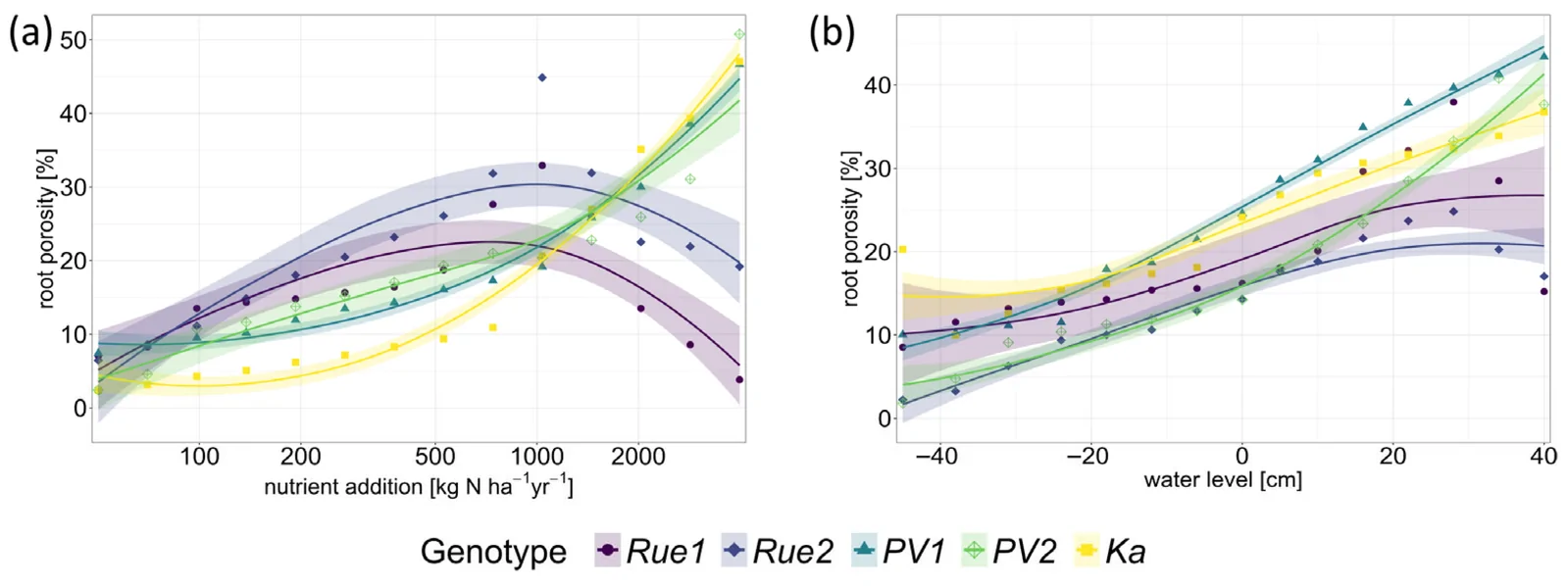
Root porosity of five *P. australis* genotypes (Rue1, Rue2, PV1, PV2, Ka; Table 1). (**a**) Root porosity [%] along the nutrient addition gradient (span = 1.5). (**b**) Root porosity [%] along the water level gradient (span = 1.4). Symbols show original data points; lines are the smoothed local polynomial regression fittings (loess). Shaded areas around lines indicate 83% confidence intervals. Along the water level gradient, negative numbers represent water levels below ground and positive numbers represent water levels above ground.

**Figure S6:**
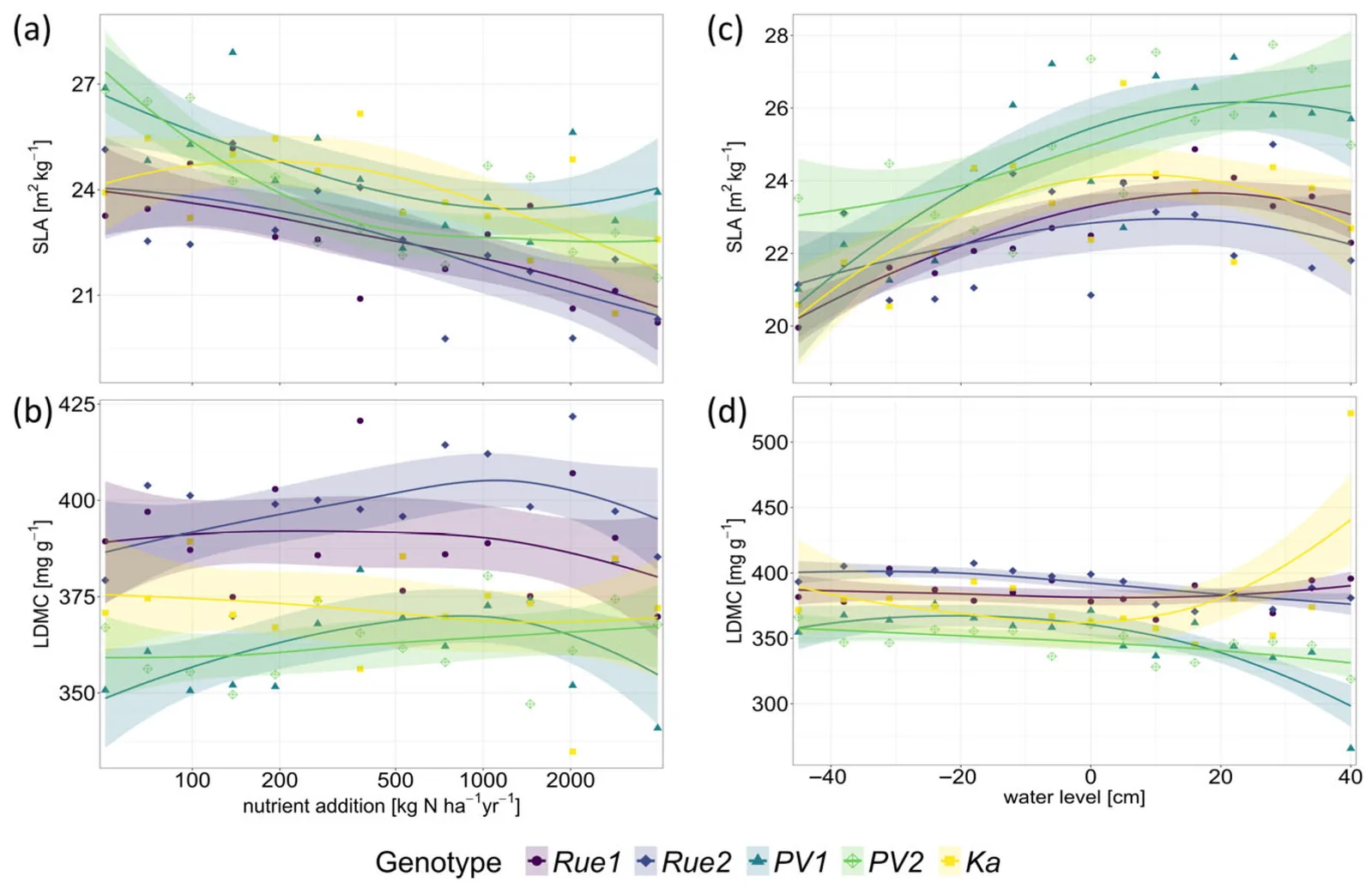
Functional leaf traits of five *P. australis* genotypes (Rue1, Rue2, PV1, PV2, Ka; Table 1). (**a**) Specific leaf area (SLA) [m^2^ kg^−1^] (span = 1.5) and (**b**) leaf dry matter content (LDMC) [mg g^−1^] (span = 1.5) along the nutrient addition gradient. (**c**) Specific leaf area (SLA) [m^2^ kg^−1^] (span = 2.0) and (**d**) leaf dry matter content (LDMC) [mg g^−1^] (span = 2.0) along the water level gradient. Symbols show original data points; lines are the smoothed local polynomial regression fittings (loess). Shaded areas around lines indicate 83% confidence intervals. Along the water level gradient, negative numbers represent water levels below ground and positive numbers represent water levels above ground.

**Figure S7:**
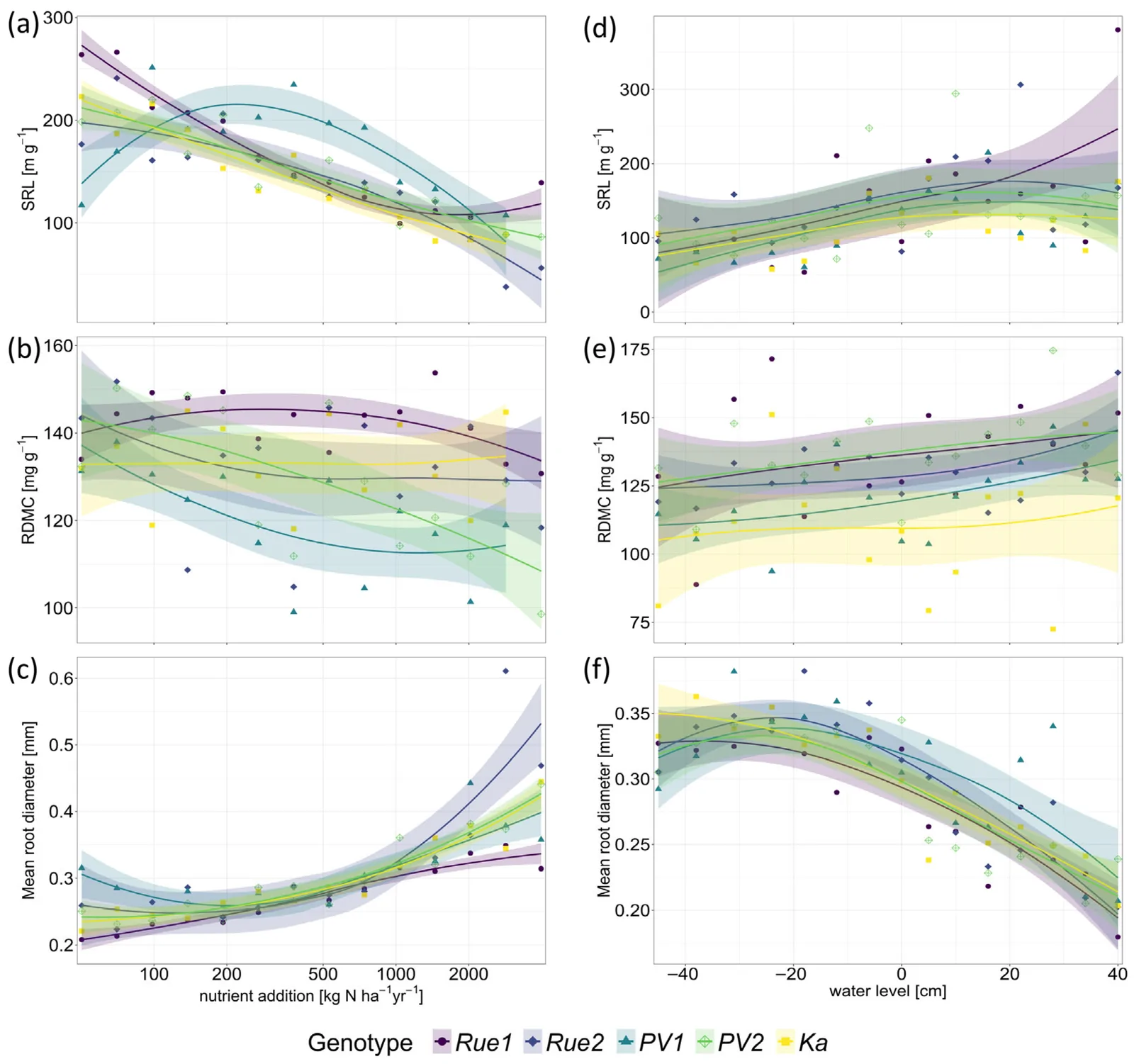
Functional root traits of five *P. australis* genotypes (Rue1, Rue2, PV1, PV2, Ka; Table 1). (**a**) Specific root length (SRL) [m g^−1^] (span = 1.2), (**b**) root dry matter content (RDMC) [mg g^−1^] (span = 2.3) and (**c**) mean root diameter [mm] (span = 1.2) along the nutrient addition gradient. (**d**) Specific root length (SRL) [m g^−1^] (span = 1.5), (**e**) root dry matter content (RDMC) [mg g^−1^] (span = 2.7) and (**f**) mean root diameter [mm] (span = 1.5) along the water level gradient. Symbols show original data points; lines are the smoothed local polynomial regression fittings (loess). Shaded areas around lines indicate 83% confidence intervals. Along the water level gradient, negative numbers represent water levels below ground and positive numbers represent water levels above ground.

**Deleted Citation**

Due to the calculation mistake described above, different N/P- and N/K-ratios than originally intended were used in the fertilization. Therefore, we corrected a sentence in Section 4. Materials and Methods, subsection 4.2 Study Design by giving the actual N/P-ratio (5.12) and N/K-ratio (0.51) and removing the citation of the references with the numbers 63 [2] and 64 [6] in the original paper. The original, but incorrect, calculation of nutrient amounts, took these sources into account. However, as we actually achieved different N/P- and N/K-ratios due to the calculation mistake described above, we removed the citation to these references in this sentence. The reference with the number 64 in the original publication was removed in the corrected manuscript. The sentence in Section 4. Materials and Methods, Section 4.2 Study Design should read: Plants were fertilized with N, phosphorus (P) and potassium (K) in the form of NH_4_NO_3_, (NH_4_)_2_HPO_4_ and K_2_CO_3_ dissolved in demineralized water (N/P = 5.12, N/K = 0.51). 

The authors state that the scientific conclusions are unaffected. This correction was approved by the Academic Editor. The original publication has also been updated.
