# Supplementary material for: Choose Wisely: Great Variation among Genotypes of Promising Paludiculture Crop Phragmites australis
Source: Plants (Basel). 2023 Feb 24;12(5):1045. doi: 10.3390/plants12051045 (PMC10004764; doi:10.3390/plants12051045)
Supplement: Supplementary file 1 [file plants-12-01045-s001.zip › plants-2201004-supplementary.pdf]

Supporting material

Supplementary information to the paper:

Haldan, K., Kuprina, K., Haase, M. I., Kieckhäfer, F., Schade, L., Schmoldt, J., Schock, L. S., Stein, M., Wille, A., Schnittler, M., Bog, M. and Kreyling, J. "Choose wisely: Great variation among genotypes of promising paludiculture crop *Phragmites australis*."

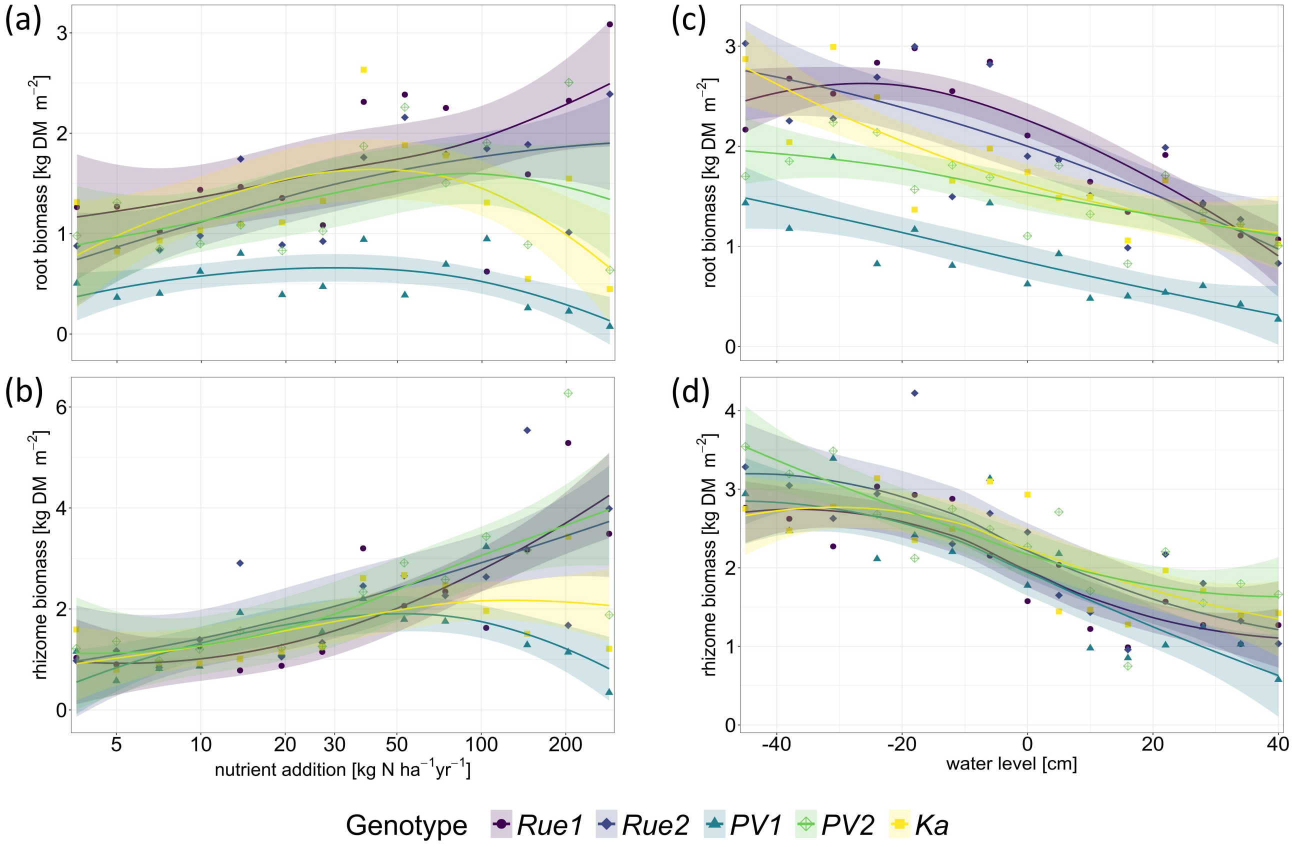

Figure S1: Root and rhizome biomass dry weight of five *P. australis* genotypes (*Rue1*, *Rue2*, *PV1*, *PV2*, *Ka*; Table 1). (**a**) Root biomass dry weight [kg m^{-2}] (span = 1.8) and (**b**) rhizome biomass dry weight [kg m^{-2}] (span = 2.0) along the nutrient addition gradient. (**c**) Root biomass dry weight [kg m^{-2}] (span = 2.0) and (**d**) rhizome biomass dry weight [kg m^{-2}] (span = 1.2) along the water level gradient. Symbols show original data points, lines are the smoothed local polynomial regression fittings (loess). Shaded areas around lines indicate 83% confidence intervals. Along the water level gradient, negative numbers represent water levels below ground, positive numbers water levels above ground.

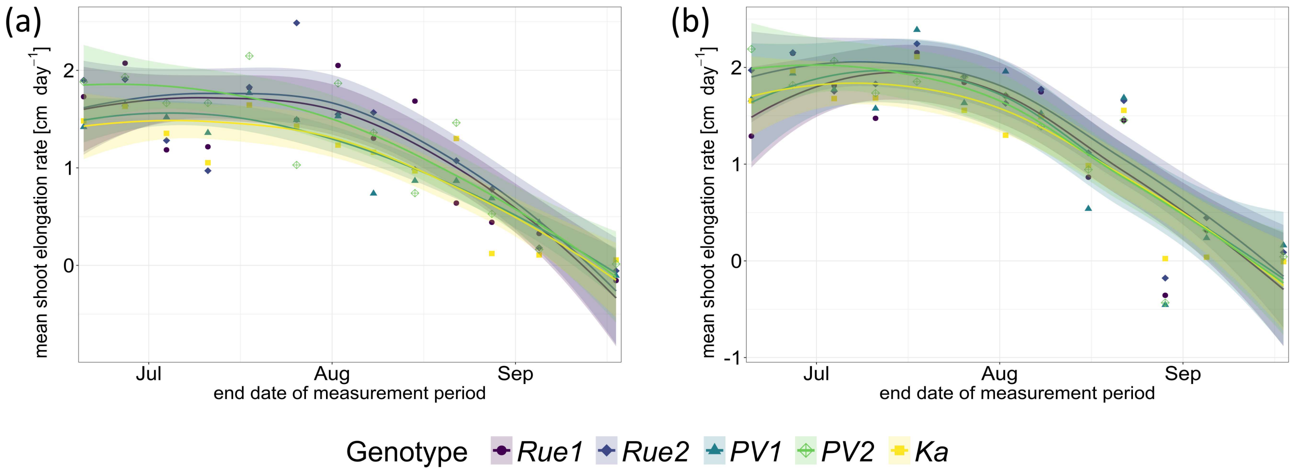

Figure S2: Shoot elongation rate of five *P. australis* genotypes (*Rue1*, *Rue2*, *PV1*, *PV2*, *Ka*; Table 1). (**a**) Shoot elongation rate [cm day^{-1}] averaged over the nutrient addition gradient (span = 1.3). (**b**) Shoot elongation rate [cm day^{-1}] averaged over the water level gradient (span = 1.2). Symbols show original data points, lines are the smoothed local polynomial regression fittings (loess). Shaded areas around lines indicate 83% confidence intervals.

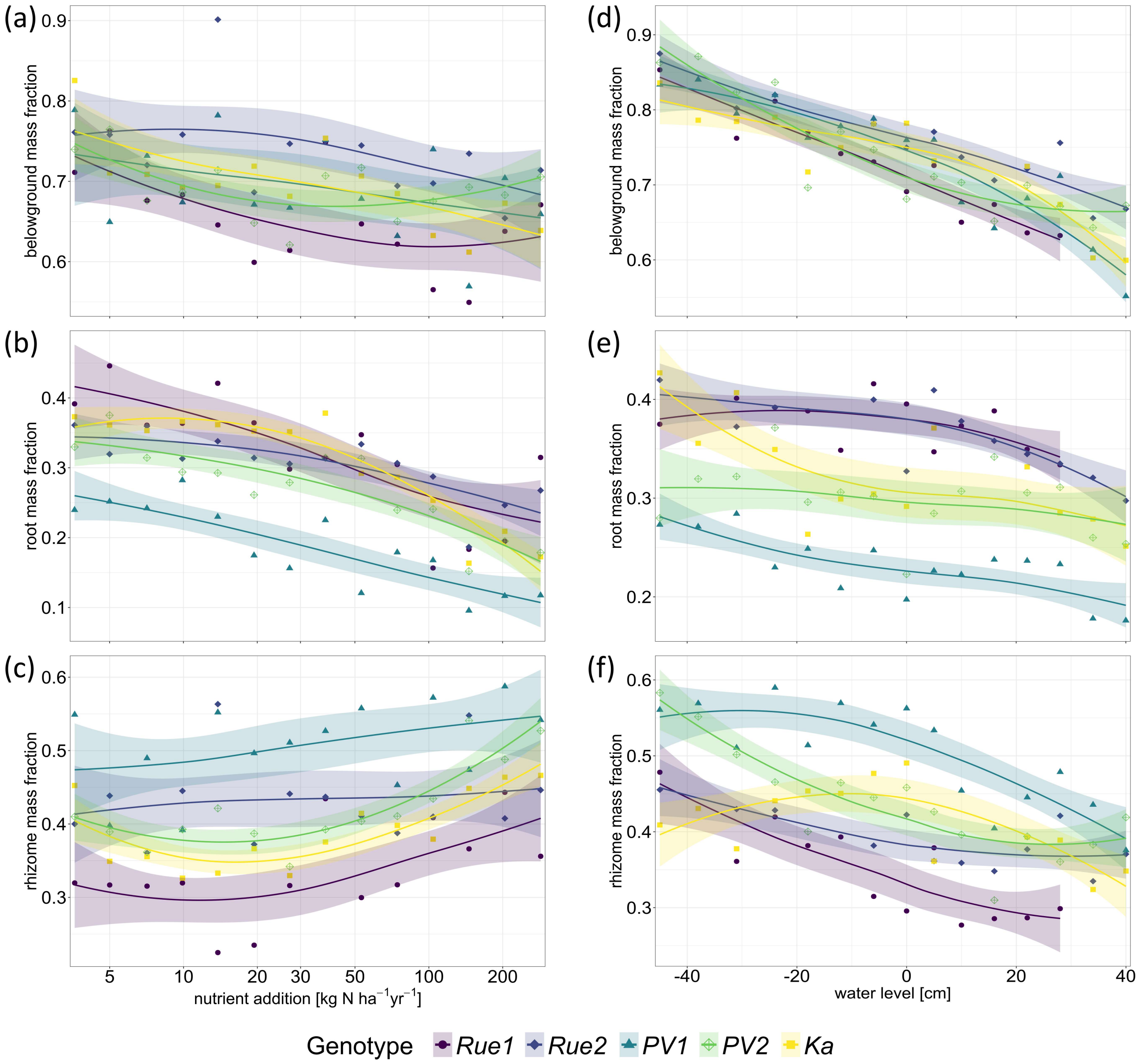

Figure S3: Biomass allocation of five *P. australis* genotypes (*Rue1*, *Rue2*, *PV1*, *PV2*, *Ka*; Table 1). (**a**) Belowground mass fraction (span = 1.8), (**b**) root mass fraction (span = 2.0) and (**c**) rhizome mass fraction (span = 1.8) along the nutrient addition gradient. (**d**) Belowground mass fraction (span = 1.2), (**e**) root mass fraction (span = 1.4) and (**f**) rhizome mass fraction (span = 1.2) along the water level gradient. Symbols show original data points, lines are the smoothed local polynomial regression fittings (loess). Shaded areas around lines indicate 83% confidence intervals. Along the water level gradient, negative numbers represent water levels below ground, positive numbers water levels above ground.

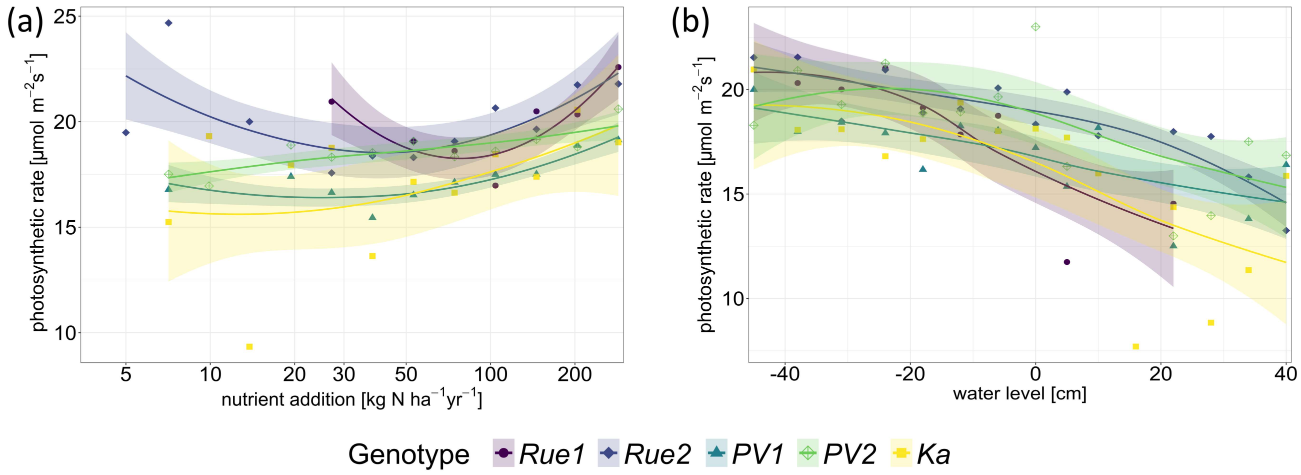

Figure S4: Photosynthetic rate of *five P. australis* genotypes (*Rue1*, *Rue2*, *PV1*, *PV2*, *Ka*; Table 1). (**a**) Photosynthetic rate [µmol m^{-2} s^{-1}] along the nutrient addition gradient (span = 2.0). (**b**) Photosynthetic rate [µmol m^{-2} s^{-1}] along the water level gradient (span = 1.5). Symbols show original data points, lines are the smoothed local polynomial regression fittings (loess). Shaded areas around lines indicate 83% confidence intervals. Along the water level gradient, negative numbers represent water levels below ground, positive numbers water levels above ground.

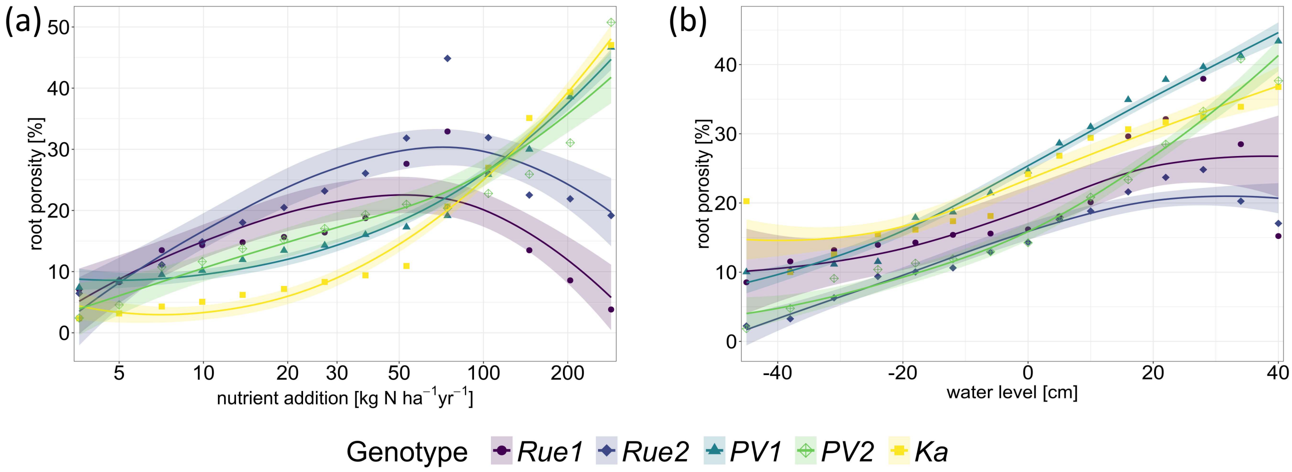

Figure S5: Root porosity of five *P. australis* genotypes (*Rue1*, *Rue2*, *PV1*, *PV2*, *Ka*; Table 1). (**a**) Root porosity [%] along the nutrient addition gradient (span = 1.5). (**b**) Root porosity [%] along the water level gradient (span = 1.4). Symbols show original data points, lines are the smoothed local polynomial regression fittings (loess). Shaded areas around lines indicate 83% confidence intervals. Along the water level gradient, negative numbers represent water levels below ground, positive numbers water levels above ground.

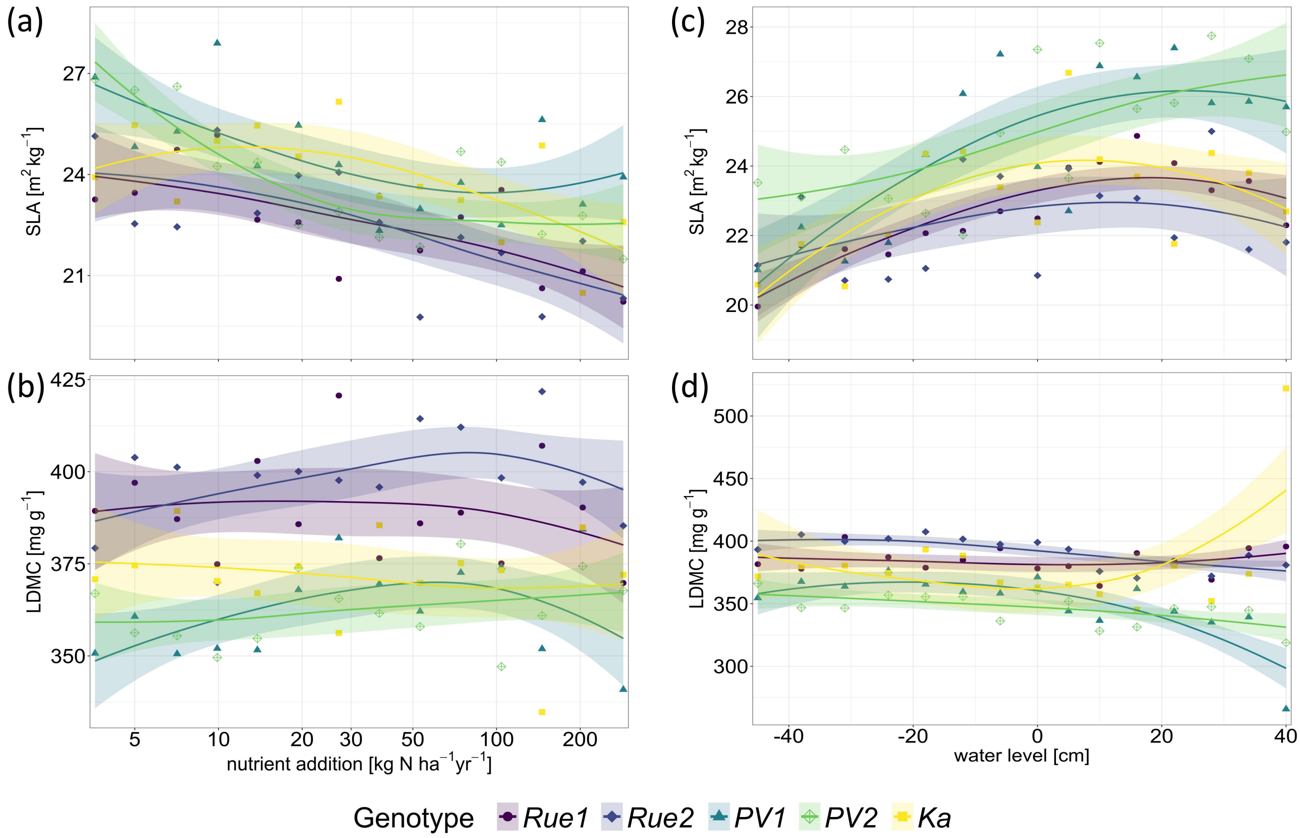

Figure S6: Functional leaf traits of five *P. australis* genotypes (*Rue1*, *Rue2*, *PV1*, *PV2*, *Ka*; Table 1). (**a**) Specific leaf area (SLA) [m^2 kg^{-1}] (span = 1.5) and (**b**) leaf dry matter content (LDMC) [mg g^{-1}] (span = 1.5) along the nutrient addition gradient. (**c**) Specific leaf area (SLA) [m^2 kg^{-1}] (span = 2.0) and (**d**) leaf dry matter content (LDMC) [mg g^{-1}] (span = 2.0) along the water level gradient. Symbols show original data points, lines are the smoothed local polynomial regression fittings (loess). Shaded areas around lines indicate 83% confidence intervals. Along the water level gradient, negative numbers represent water levels below ground, positive numbers water levels above ground.

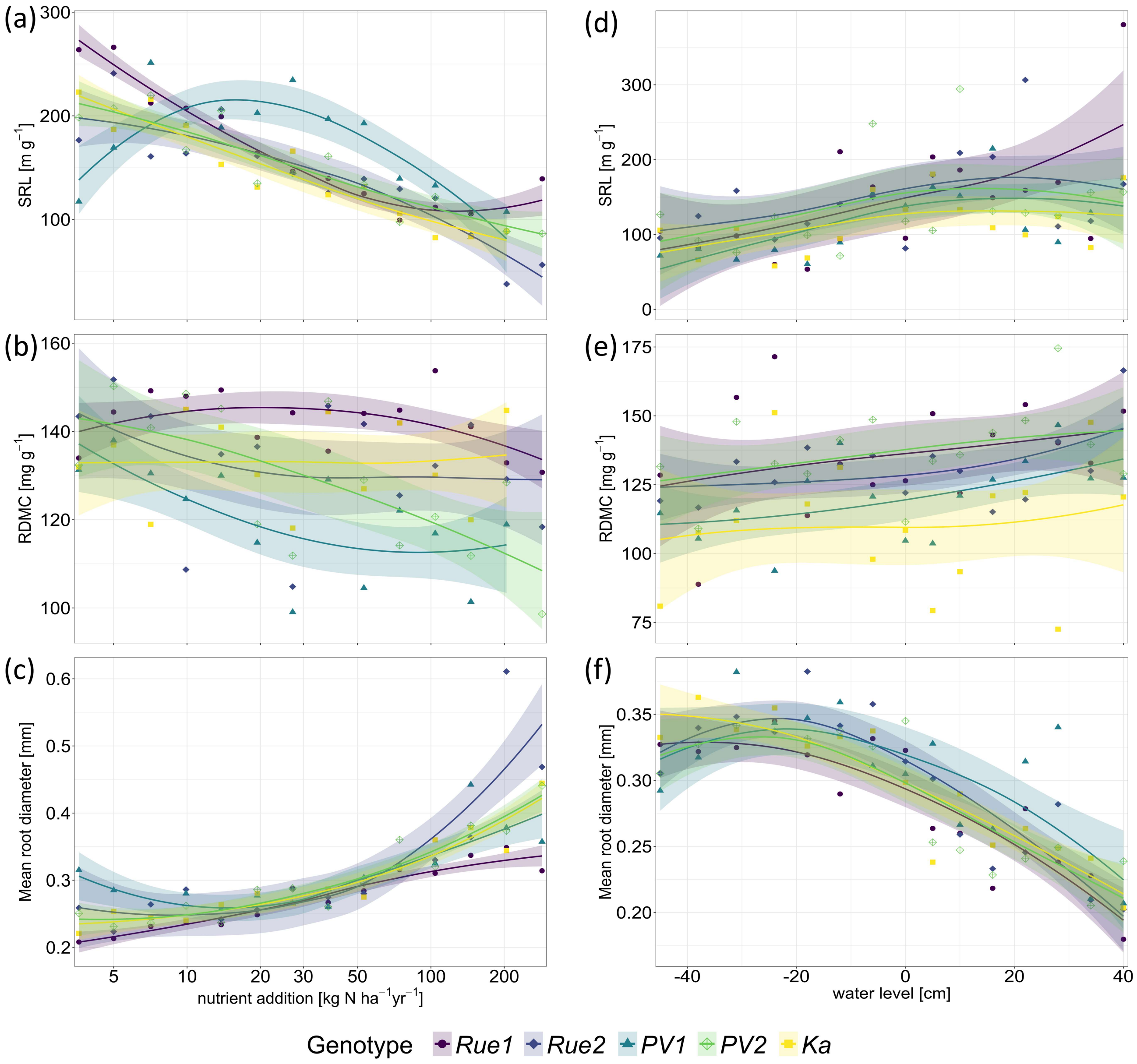

Figure S7: Functional root traits of five *P. australis* genotypes (*Rue1*, *Rue2*, *PV1*, *PV2*, *Ka*; Table 1). (**a**) Specific root length (SRL) [m g^{-1}] (span = 1.2), (**b**) root dry matter content (RDMC) [mg g^{-1}] (span = 2.3) and (**c**) mean root diameter [mm] (span = 1.2) along the nutrient addition gradient. (**d**) Specific root length (SRL) [m g^{-1}] (span = 1.5), (**e**) root dry matter content (RDMC) [mg g^{-1}] (span = 2.7) and (**f**) mean root diameter [mm] (span = 1.5) along the water level gradient. Symbols show original data points, lines are the smoothed local polynomial regression fittings (loess). Shaded areas around lines indicate 83% confidence intervals. Along the water level gradient, negative numbers represent water levels below ground, positive numbers water levels above ground.
